# Functional Effects of Cardiomyocyte Injury in COVID-19

**DOI:** 10.1128/JVI.01063-21

**Published:** 2022-01-26

**Authors:** Mustafa M. Siddiq, Angel T. Chan, Lisa Miorin, Arjun S. Yadaw, Kristin G. Beaumont, Thomas Kehrer, Anastasija Cupic, Kris M. White, Rosa E. Tolentino, Bin Hu, Alan D. Stern, Iman Tavassoly, Jens Hansen, Robert Sebra, Pedro Martinez, Som Prabha, Nicole Dubois, Christoph Schaniel, Rupa Iyengar-Kapuganti, Nina Kukar, Gennaro Giustino, Karan Sud, Sharon Nirenberg, Patricia Kovatch, Randy A. Albrecht, Joseph Goldfarb, Lori Croft, Maryann A. McLaughlin, Edgar Argulian, Stamatios Lerakis, Jagat Narula, Adolfo García-Sastre, Ravi Iyengar

**Affiliations:** a Department of Pharmacological Sciences, Icahn School of Medicine at Mount Sinai, New York, New York, USA; b Institute for Systems Biomedicine, Icahn School of Medicine at Mount Sinai, New York, New York, USA; c Department of Medicine and Radiology, Memorial Sloan Kettering Cancer Center, New York, New York, USA; d Department of Microbiology, Icahn School of Medicine at Mount Sinai, New York, New York, USA; e Global Health and Emerging Pathogens Institute, Icahn School of Medicine at Mount Sinai, New York, New York, USA; f Department of Genetics and Genomic Sciences, Icahn School of Medicine at Mount Sinai, New York, New York, USA; g Department of Cell Developmental and Regenerative Biology, Icahn School of Medicine at Mount Sinai, New York, New York, USA; h Black Family Stem Cell Center, Icahn School of Medicine at Mount Sinai, New York, New York, USA; i Division of Hematology & Oncology, Department of Medicine, Icahn School of Medicine at Mount Sinai, New York, New York, USA; j Division of Cardiology, Department of Medicine, Icahn School of Medicine at Mount Sinai, New York, New York, USA; k Department of Scientific Computing and Data Science, Icahn School of Medicine at Mount Sinai, New York, New York, USA; l Department of Medicine, Division of Infectious Diseases, Icahn School of Medicine at Mount Sinai, New York, New York, USA; m Tisch Cancer Institute, Icahn School of Medicine at Mount Sinai, New York, New York, USA; n Graduate School of Biomedical Sciences, Icahn School of Medicine at Mount Sinai, New York, New York, USA; o Icahn Institute for Data Science and Genomic Technology, Icahn School of Medicine at Mount Sinai, New York, New York, USA; p Sema4, a Mount Sinai Venture, Stamford, Connecticut, USA; Loyola University Chicago

**Keywords:** COVID-19, IL-10, IL-1β, IL-6, machine learning, NL63, SARS-CoV-2, cardiac disease, cardiomyocytes, computational biology

## Abstract

COVID-19 affects multiple organs. Clinical data from the Mount Sinai Health System show that substantial numbers of COVID-19 patients without prior heart disease develop cardiac dysfunction. How COVID-19 patients develop cardiac disease is not known. We integrated cell biological and physiological analyses of human cardiomyocytes differentiated from human induced pluripotent stem cells (hiPSCs) infected with severe acute respiratory syndrome coronavirus 2 (SARS-CoV-2) in the presence of interleukins (ILs) with clinical findings related to laboratory values in COVID-19 patients to identify plausible mechanisms of cardiac disease in COVID-19 patients. We infected hiPSC-derived cardiomyocytes from healthy human subjects with SARS-CoV-2 in the absence and presence of IL-6 and IL-1β. Infection resulted in increased numbers of multinucleated cells. Interleukin treatment and infection resulted in disorganization of myofibrils, extracellular release of troponin I, and reduced and erratic beating. Infection resulted in decreased expression of mRNA encoding key proteins of the cardiomyocyte contractile apparatus. Although interleukins did not increase the extent of infection, they increased the contractile dysfunction associated with viral infection of cardiomyocytes, resulting in cessation of beating. Clinical data from hospitalized patients from the Mount Sinai Health System show that a significant portion of COVID-19 patients without history of heart disease have elevated troponin and interleukin levels. A substantial subset of these patients showed reduced left ventricular function by echocardiography. Our laboratory observations, combined with the clinical data, indicate that direct effects on cardiomyocytes by interleukins and SARS-CoV-2 infection might underlie heart disease in COVID-19 patients.

**IMPORTANCE** SARS-CoV-2 infects multiple organs, including the heart. Analyses of hospitalized patients show that a substantial number without prior indication of heart disease or comorbidities show significant injury to heart tissue, assessed by increased levels of troponin in blood. We studied the cell biological and physiological effects of virus infection of healthy human iPSC-derived cardiomyocytes in culture. Virus infection with interleukins disorganizes myofibrils, increases cell size and the numbers of multinucleated cells, and suppresses the expression of proteins of the contractile apparatus. Viral infection of cardiomyocytes in culture triggers release of troponin similar to elevation in levels of COVID-19 patients with heart disease. Viral infection in the presence of interleukins slows down and desynchronizes the beating of cardiomyocytes in culture. The cell-level physiological changes are similar to decreases in left ventricular ejection seen in imaging of patients’ hearts. These observations suggest that direct injury to heart tissue by virus can be one underlying cause of heart disease in COVID-19.

## INTRODUCTION

Severe acute respiratory syndrome coronavirus 2 (SARS-CoV-2) infections have created a global COVID-19 pandemic that continues unabated in 2021. With over 170 million cases worldwide (1), COVID-19 has affected people in every country. This is in contrast to other coronavirus outbreaks such as SARS (2) and Middle East respiratory syndrome (MERS) (3) that were geographically limited. Significant numbers of patients with COVID-19 display multiorgan pathophysiology. There is substantial evidence that a significant proportion of COVID-19 patients develop both cardiac (4–6) and kidney (7, 8) dysfunction. Such multiorgan pathophysiology is perhaps not entirely surprising since the receptor for SARS-CoV-2 is the angiotensin-converting enzyme 2 (ACE2) protein (9), which is widely expressed in many tissues, including the heart and kidney (10).

Myocardial injury associated with COVID-19 has been observed in substantial numbers of patients (11). Biomarkers of injury include elevated troponin and B-type natriuretic peptide (BNP) protein levels in blood (12) and abnormal echocardiography (13, 14). The reasons for the myocardial injury remain unclear, and autopsy results have shown that COVID-19 patients show sparse infection of cardiac tissue and do not show the typical hallmarks of viral myocarditis (15). Myocardial injury can have multiple causes, including formation of microthrombi that have been observed in COVID-19 patients (16) and oxygen insufficiency due to pulmonary disease, which is commonly observed and in our analyses is an important predictor of mortality (17). Another feature observed in the clinical data is that most COVID-19 patients have elevated interleukins (ILs) (18). Interleukins are known to affect cardiomyocyte (CM) function (19). Multiple reports have indicated that human CMs can be infected by SARS-CoV-2 (20–22). In this study, we integrated findings from analyses of fully anonymized clinical data on COVID-19 patients from the Mount Sinai Health System with those from studies on human induced pluripotent stem cell (hiPSC)-derived ventricular cardiomyocytes in culture infected with SARS-CoV-2 in the absence and presence of physiological concentrations of IL-6 and IL-1β. We demonstrate that changes in cardiomyocyte cell biology, including myofibril organization and decreased expression of key proteins of the cardiomyocyte contractile apparatus, and in physiology, such as release of troponin and erratic beating of cardiomyocytes, can account for key features of myocardial injury observed in patients. These observations demonstrate that direct injury by virus to cardiac tissue during COVID-19 can play a role in decreased cardiac function.

## RESULTS

### COVID-19 patients without prior heart disease show elevated troponin levels.

**FIG 1:**
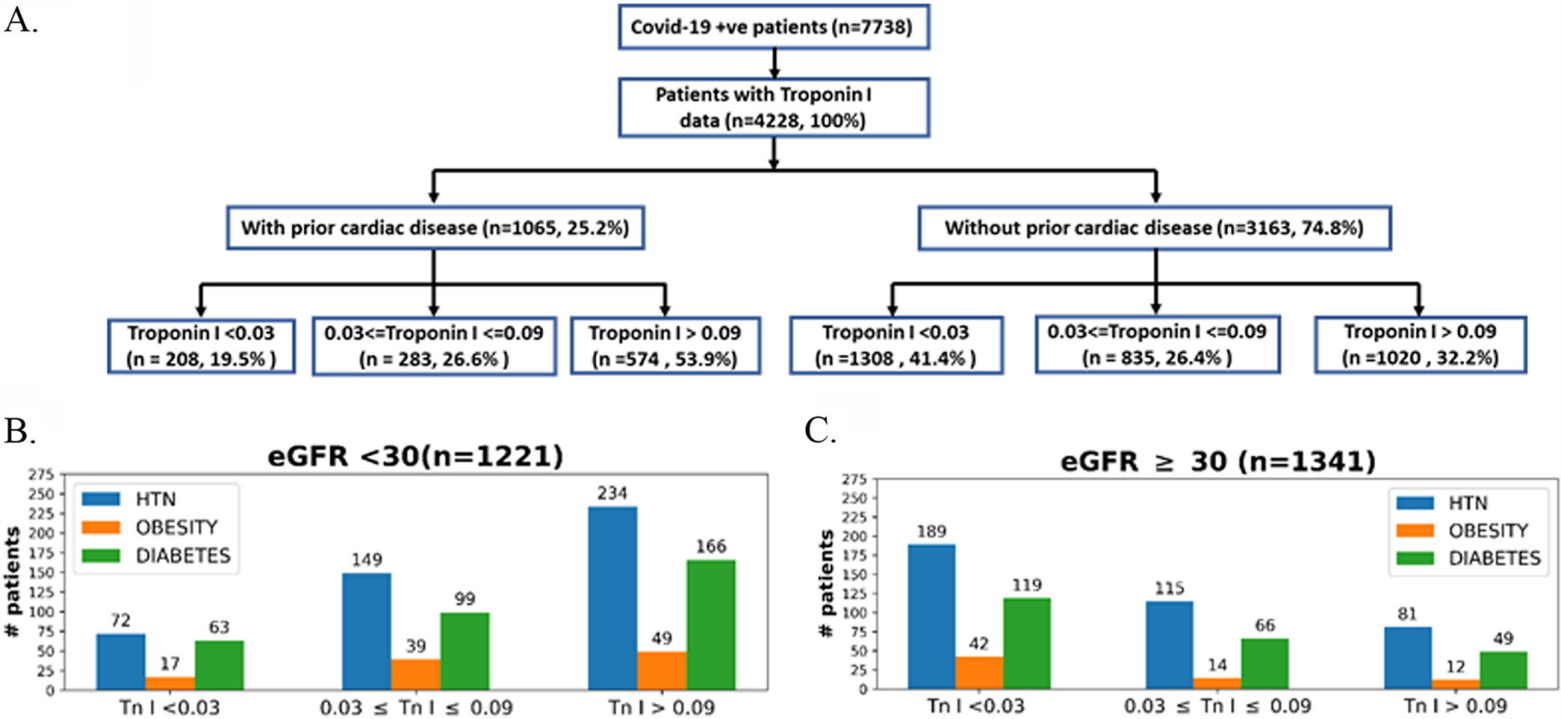
COVID-19 patients without history of cardiac disease have elevated troponin I levels and other clinical characteristics associated with cardiac dysfunction. Shown are clinical data from COVID-19-positive patients (with encounter data, labs, and vital signs; *n* = 7,738) downloaded from the Mount Sinai Data Warehouse on 15 July 2020. (A) Flowchart for COVID-19 patients for whom troponin I measurements were available (*n* = 4,228). These patients were further divided into patients with and without prior cardiac disease. Patients having one or more of the three comorbidities or a history of (i) coronary artery disease, (ii) atrial fibrillation, or (iii) heart failure were binned as “with prior cardiac disease.” Each category was further divided into subgroups with respect to troponin levels using standard clinical cutoffs. A total of 32.2% (1,020/3,163) of COVID-19 patients without a history of heart disease have clinically significant elevated levels (>0.09 ng/mL) of troponin I. Patients without prior cardiac disease were classified with respect to kidney function using eGFR values of <30 (B) or ≥30 (C) and binned for comorbidities (hypertension [HTN], obesity, or diabetes) in the three cohorts with different troponin I levels.

**FIG 2:**
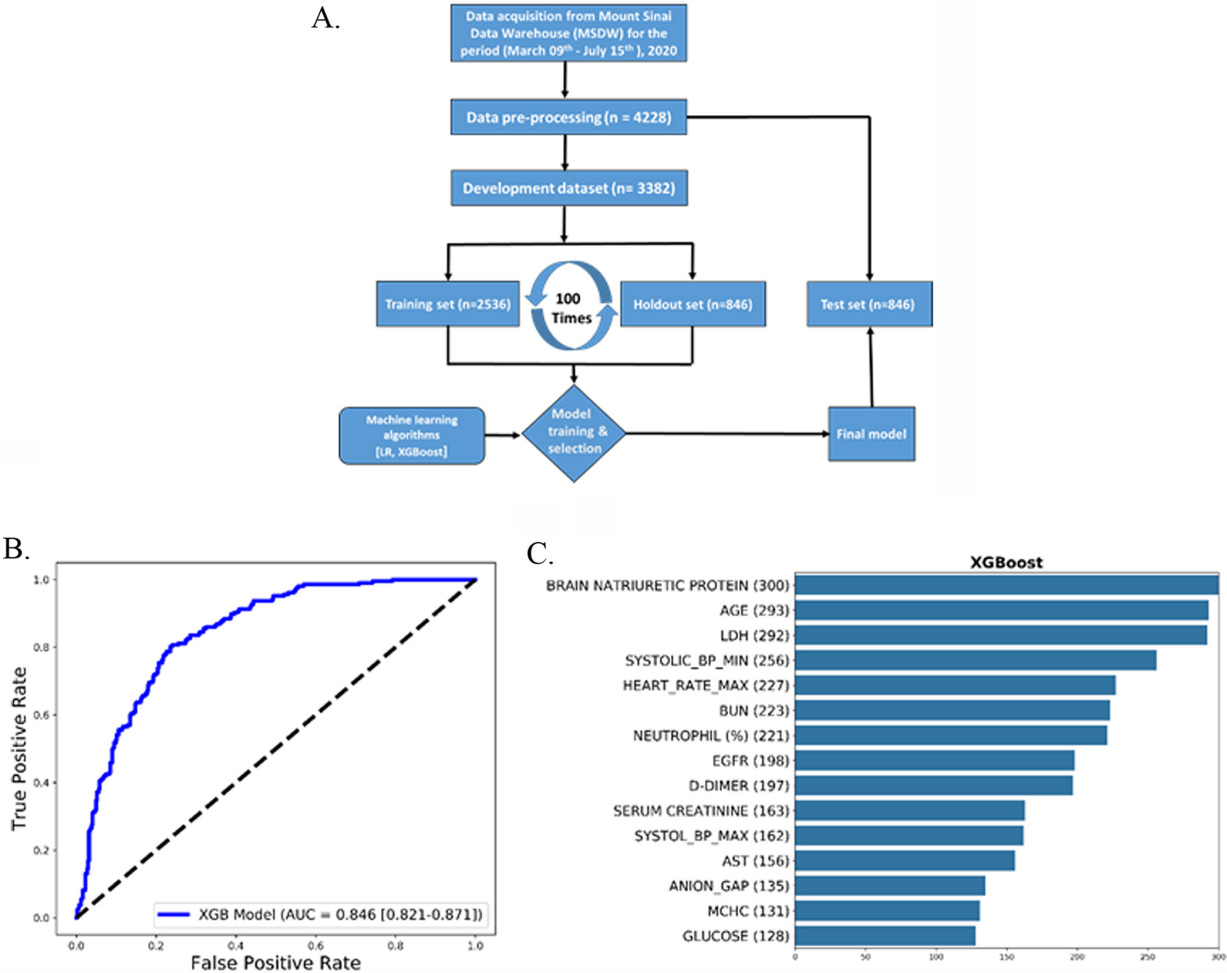
Predictive machine learning models to identify clinical features that predict elevated levels of troponin I. (A) We developed predictive machine learning models to identify clinical features that predict elevated levels of troponin I. The workflow for model development is shown in panel A. After preprocessing, data for patients with COVID-19 with troponin I data (*n* = 4,228) were randomly divided in an 80:20 ratio into a prediction model development data set (*n* = 3,382) and an independent retrospective validation data set (test data set; *n* = 846). For prediction model training and selection, the development data set was further randomly split into a 75% training data set (*n* = 2,536) and a 25% holdout data set (*n* = 846). We ran an imputation model on the training set to obtain an optimum missing value imputation cutoff, which is 35% for this model (Fig. S1). We used a recursive feature elimination method to obtain the optimum number of features to reach a plateau (Fig. S2). We tested two classification algorithms, and the XGBoost (eXtreme Gradient Boosting) classification algorithm performed better than logistic regression (Fig. S3). The final predictive model was validated on the test data set (*n* = 846). (B) Evaluation results for the test set are shown in terms of the ROC curves obtained, as well as their AUC scores, with the 95% confidence interval in parentheses. (C) The top 15 predictive features identified using the recursive feature elimination method for XGBoost classification algorithms across the three independent sets of 100 runs used to select the most discriminative features and train the corresponding candidate prediction models. The values in parentheses indicate the number of times the feature was selected as top ranked in the development data set.

During the early phase of the pandemic in New York City in spring 2020, substantial numbers of patients were over 55 years old (23). This was also true of patients in the Mount Sinai Health System (see Table S1 in the supplemental material). Hence, we parsed the deidentified clinical data compiled by the Mount Sinai Data Warehouse (MSDW) to identify COVID-19 patients with and without history of heart disease. For this, we used three comorbidities—coronary artery disease, atrial fibrillation, and heart failure—listed in the MSDW data for each patient as prior indications of heart disease. We parsed the COVID-19 patients to determine the size of the subset that did not have prior heart disease but did show cardiac dysfunction during COVID-19. We used elevated troponin I levels in blood as a measure of cardiac dysfunction (24). Of these patients, 32.25% (1,020/3,163) show elevated blood troponin levels (Fig. 1A). Since elevated blood troponin levels can occur due to kidney dysfunction, patients were binned with an estimated glomerular filtration rate (eGFR) of <30 mL/min or ≥30 mL/min. A total of 21% (281/1341) of the patients with an eGFR of ≥30 mL/min (patients whose kidney function is not diminished) showed elevated levels of blood troponin (Fig. 1B). Some of these 281 patients had comorbidities that are indicated in Fig. 1B. The overall numbers indicate 44% of patients (901/2562) of COVID-19 patients who have recorded eGFR values in MSDW have diminished kidney function (eGFR, <30) (Fig. 1B). Comorbidities for some of the patients at different levels of troponin are shown in Fig. 1C. Overall, we find a significant number of COVID-19 patients with normal kidney function show elevated blood troponin I levels. In order to further verify that blood troponin I levels represent cardiac dysfunction, we sought to determine, in an unbiased manner, other clinical features associated with elevated troponin I in COVID-19 patients. For this, we used machine learning models to predict the top-ranked features in patients with elevated troponin I levels. We had previously used this approach to predict clinical indicators of probable mortality in COVID-19 patients (17). The flowchart in Fig. 2A shows how we developed and used the machine learning model (57). The XGBoost algorithm model gave good predictive performance, with an AUC (area under the curve) of 0.846 (Fig. 2B). Details of model comparison and performance are shown in Fig. S1 to S3 and Tables S1 and S2 in the supplemental material. We used the XGBoost model to identify the top-ranked features that predict elevated blood troponin. The list is shown in Fig. 2. It can be seen that three of the top five features BNP, systolic blood pressure minimum, and heart rate directly relate to cardiac function. We conclude that release of troponin can be used to evaluate cardiomyocyte injury upon infection with SARS-CoV-2.

### Cardiomyocytes from hiPSCs when infected with SARS-CoV-2 in the presence of ILs are damaged and release troponin.

**FIG 3:**
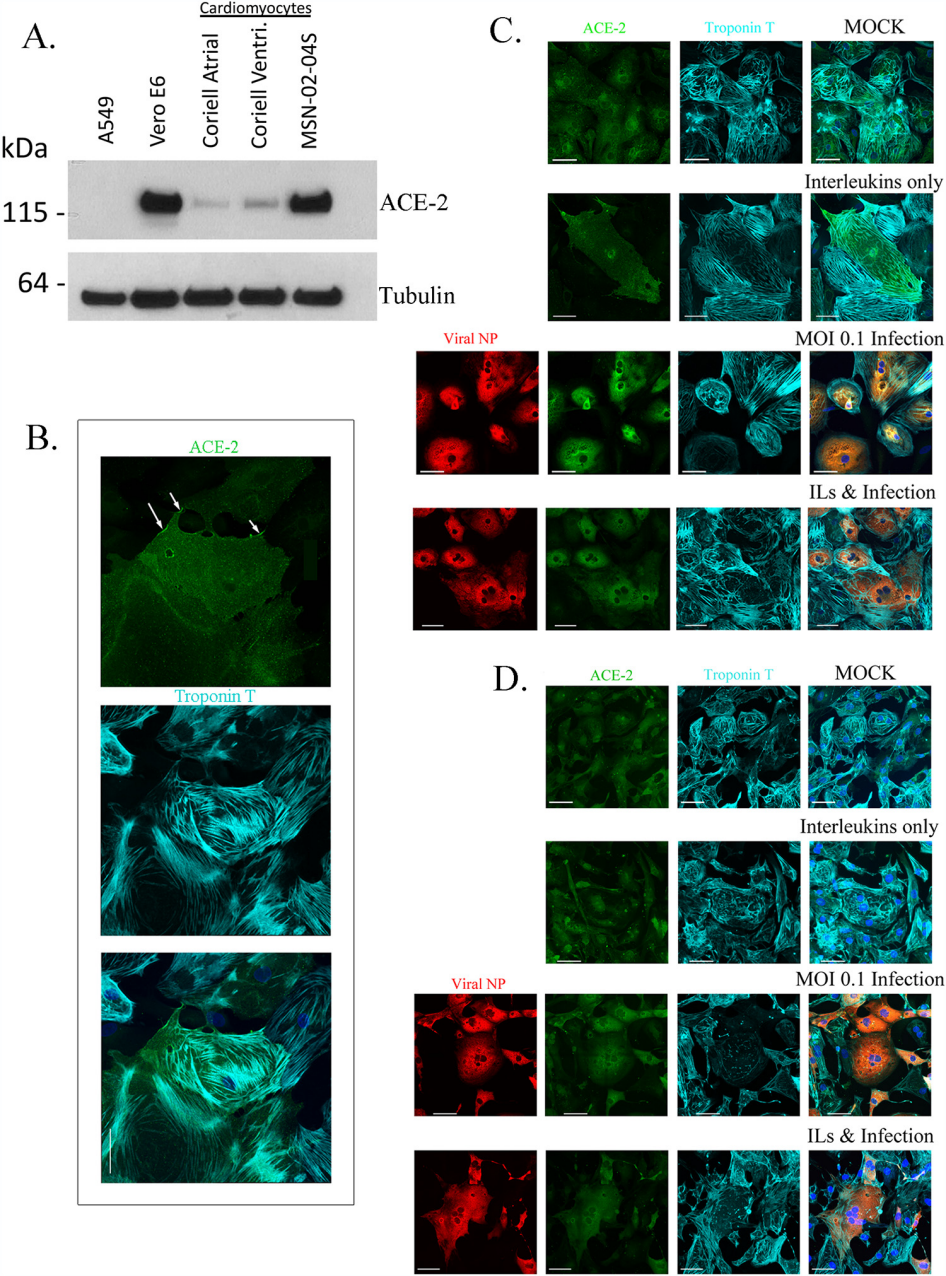
Characterization of hiPSC-derived ventricular cardiomyocytes infected by SARS-CoV-2. hiPSC cells reprogrammed from skin fibroblasts of healthy subjects were differentiated into beating ventricular cardiomyocytes (CMs) and after culture for 30 days were used in these experiments. (A) Expression of ACE 2 protein. By immunoblotting, the expression of ACE2 was identified in cardiomyocytes. For negative controls, we used A549 cells, which had no detectable levels of ACE2. Vero E6 cells are positive controls for ACE2 expression, as confirmed using R&D antibody AF933. Three different human iPSC-derived cardiomyocytes were tested: two were from the Coriell Institute for Medical Research (Actin2 EGFR D60 atrial and ventricular [Ventri.]), and one was from the LINCS Institute (MSN-02-04S). (B) Immunostaining of CMs for ACE2 (green) and troponin T (cyan). Although all cells express ACE2, we find only some cells express ACE2 on the plasma membrane, as indicated by the arrows (top panel). The CMs express troponin T (cyan), which is seen as ordered myofibrils (middle panel). The image is a z-stack of 8 slices that was made into a maximum-intensity projection. Overlay of the two stains is shown in the bottom panel. The scale bar in the composite image is 50 μm. (C and D) The top panels show mock-infected cardiomyocytes express ACE2 and troponin T. Nuclei are stained by Hoechst stain. The level 2 panels show that CMs treated with 30 ng/ml each of IL-6 and IL-1β express ACE2, the cells appear larger, and the troponin T appears more disorganized. The level 3 panels show CMs can be infected with SARS-CoV-2 at an MOI of 0.1, as detected by positive immunostaining for viral NP (red). All infected cells were positive for ACE2, with some cells having notable amounts of troponin T disruption. The level 4 panels show that cells infected with SARS-CoV-2 in the presence of ILs also show increase in cell diameter and disruption in troponin T organization. The scale bar in the composite image is 50 μm. Panel C shows CM line MSN31-01S. Panel D shows CM line MSN08-06S in a repeat of the experiment shown in panel C, and the cells shown are the CMs beating in Movie S1 at https://iyengarlab.org.

We have developed a library of hiPSCs from a racially/ethnically diverse population of healthy subjects (25). Since the clinical data analyses in Fig. 1 and 2 indicate that COVID-19 patients without prior indication of heart disease can develop cardiac dysfunction, we used hiPSCs from volunteers who had been clinically classified as healthy by three physicians using a variety of criteria, including electrocardiograms (25), to obtain ventricular cardiomyocytes for the cell biological and physiological analyses. Differentiated ventricular cardiomyocytes from multiple hiPSC lines were used in these studies. In all cases, we obtained near-homogeneous cardiomyocyte populations by lactate selection (26). In these hiPSC-derived CMs, we first determined the expression of ACE2, the SARS-CoV-2 receptor, by immunoblotting. We compared the CMs to Vero E6 cells, an African green monkey kidney cell line known to express ACE2 and to support robust replication of SARS-CoV-2 (Fig. 3A). We also had a negative control for ACE2 expression using A549, a cell line known to not express ACE2. We confirmed that 3 different lines of CMs have the predicted 120-kDa band when probed for with an antibody against ACE2 (Fig. 3A). By immunohistochemistry, we confirmed the expression of ACE2 in the CMs, which was predominately cytoplasmic, but in some cells was detected on the cell surface. Membrane expression is highlighted by arrows in Fig. 3B, top panel. We confirmed that the cells are indeed CMs by immunostaining for troponin T, a known marker for myofibrils (27) (Fig. 3B, middle panel). An overlay of the ACE2 and troponin staining is shown in the bottom panel of Fig. 3B. In MSDW, 2,235 patients out of the 4,228 patients with troponin I values also had IL-6 values. Within this group, 84% of COVID-19 patients (1,872/2,235) have elevated IL-6 values of 30 pg/mL (18), as would be expected for patients with an active infection, but it was not uncommon to find patients with IL-6 values that were 10^3^-fold higher than that and has been observed in patients with Andes virus (28) and in COVID-19 patients (29). Numerous interleukins have been shown to be elevated in COVID-19 patients (18, 29). However, since IL-6 values in blood are most commonly available in clinical data sets, we focused on the ILs IL-6 and IL-1β. We chose a concentration of 10 to 30 ng/mL ILs to utilize in our *in vitro* studies based on published reports of other studies in CM lines and treatment with ILs (30).

We hypothesized that studying SARS-CoV-2 infection of CMs in the presence of ILs would better approximate what myocardial tissue encounters *in vivo*. Hence, we tested the effects of SARS-CoV-2 infection on CMs in the absence and presence of ILs in the culture medium. Compared to the controls (mock-infected cells in the absence of ILs), the CMs treated with IL-6 and IL-1β appeared larger in diameter, and the myofibrils immunostained with troponin T were disorganized in two different CM lines (Fig. 3C and D, compare top and 2nd-level panels). Infection with SARS-CoV-2 at a multiplicity of infection (MOI) of 0.1 alone also appeared to increase the cell diameter compared to mock-infected controls (3rd-level panels). Infection in the presence of ILs produced the maximal effects, as assessed by the apparent increase in cell size and disorganized myofibrils (Fig. 3C and D, bottom panels). SARS-CoV-2 at an MOI of 0.1 for 48 h can infect CMs, as confirmed by immunostaining for viral nucleocapsid protein (NP) (Fig. 3C and D, red staining in the leftmost two panels). All the infected cells were positive for ACE2 (green stain) and troponin T (cyan stain), and the myofibrils were disorganized, as indicated by the troponin T staining (Fig. 3C and D, lower panels).

**FIG 4:**
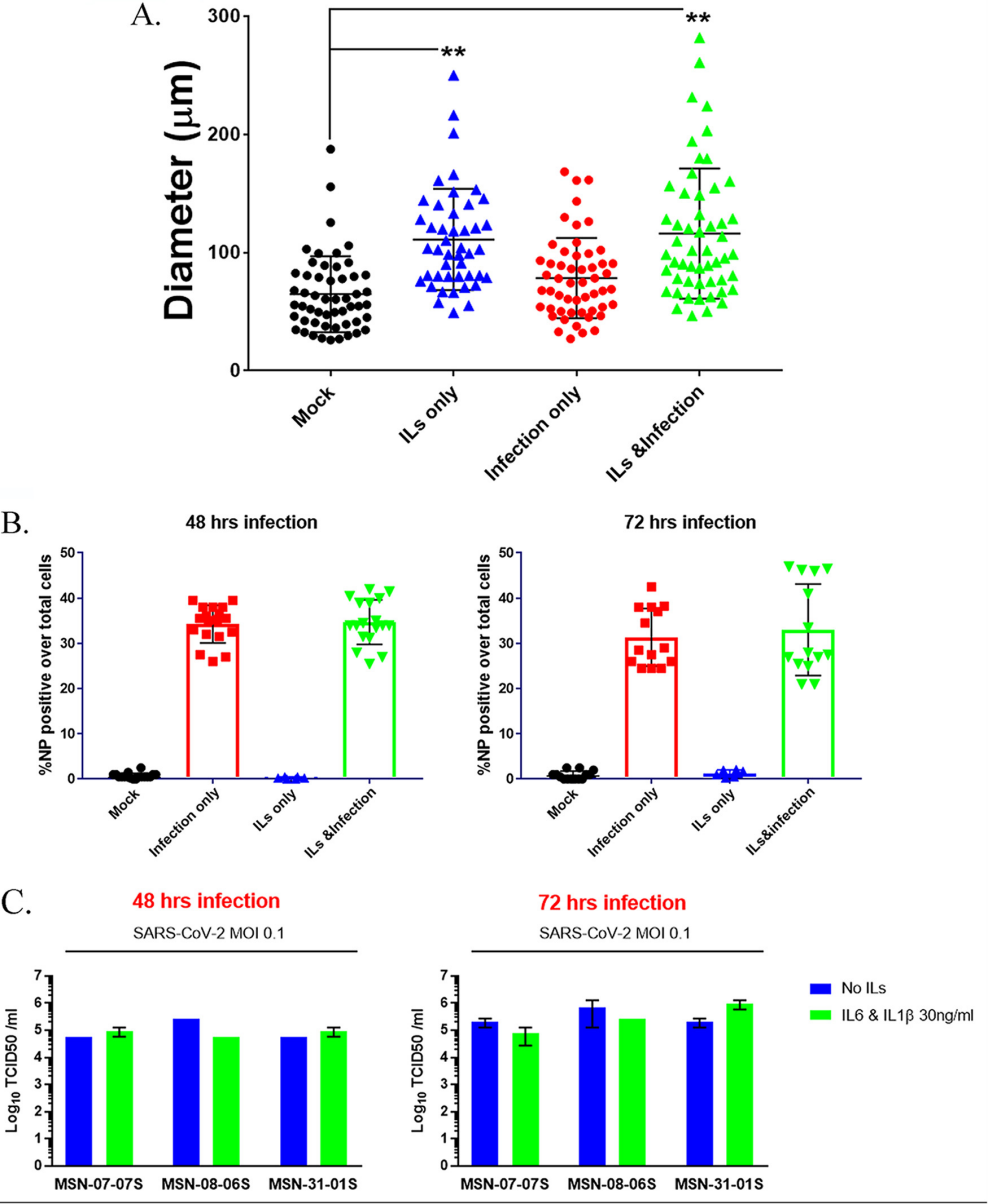
IL-6 and IL-1β can increase cell diameter but do not affect infectivity of SARS-CoV-2. (A) The changes in cell diameter with IL treatment and/or infection were measured using ImageJ. Cell diameters for two different CM lines were measured and plotted. We observe a statistically significant increase in cell size with infection but a larger increase in cell diameter with ILs only or ILs and infection with SARS-CoV-2. Statistical significance was determined by one-way ANOVA with Bonferroni multiple-comparison test: *, *P* < 0.05; **, *P* < 0.01. (B) We counted the numbers of cells stained by NP protein antibody as a measure of infectivity. Each point is representative of one well of a 96-well plate with 10,000 CM cells. The bar graph is the average of four different CM lines measuring viral NP protein-positive cells over the total number of cells. Counting was done in an automated fashion using the IN Cell Analyzer. ILs do not increase the percentage of CM cells infected by SARS-CoV-2. (C) To confirm that CMs were being productively infected and shedding SARS-CoV-2 into the culture medium, we collected supernatants from 3 different CM lines 48 and 72 h postinfection, with or without ILs, and performed a TCID_50_ plaque assay. We observed that the CM lines were infected, and the level of infection, as assessed by virus release into culture medium, did not increase with the addition of IL-6 and IL-1β.

To determine if the observed increases in cell size were statistically meaningful, we randomly measured cellular diameter in CMs without and with infection and treatment with ILs at >50 cells/per condition from two different CM lines using ImageJ and found that interleukins alone significantly increased the cell diameter. Infection alone did not significantly increase the cell diameter compared to that of mock-infected controls, but there was in increase in cell diameter in the presence of ILs (Fig. 4A). We also tested whether interleukins increased the percentage of cells infected by SARS-CoV-2. We stained the cells for viral NP and Hoechst nuclear stain to count total cell numbers and cells that stained for NP protein (Fig. 4B). We used the IN Cell Analyzer to automate the cell count for percentage of cells positive for NP. At both 48 and 72 h postinfection, ILs did not alter the level of infection of CMs by SARS-CoV-2 (Fig. 4C; see the expanded version in Fig. S4A in the supplemental material). From these cells, we collected the medium to determine the production and shedding of virus. We collected supernatants from infected CMs using three different lines (MSN-07-07S, MSN-08-06S, and MSN-31-01S) and performed analysis by 50% tissue culture infective dose (TCID_50_ [i.e., an assay to quantitate viral infections]) at 48 and 72 h postinfection (Fig. 4C; see the expanded version in Fig. S4B). We observed that all three CM lines were productively infected using an MOI of 0.1.

**FIG 5:**
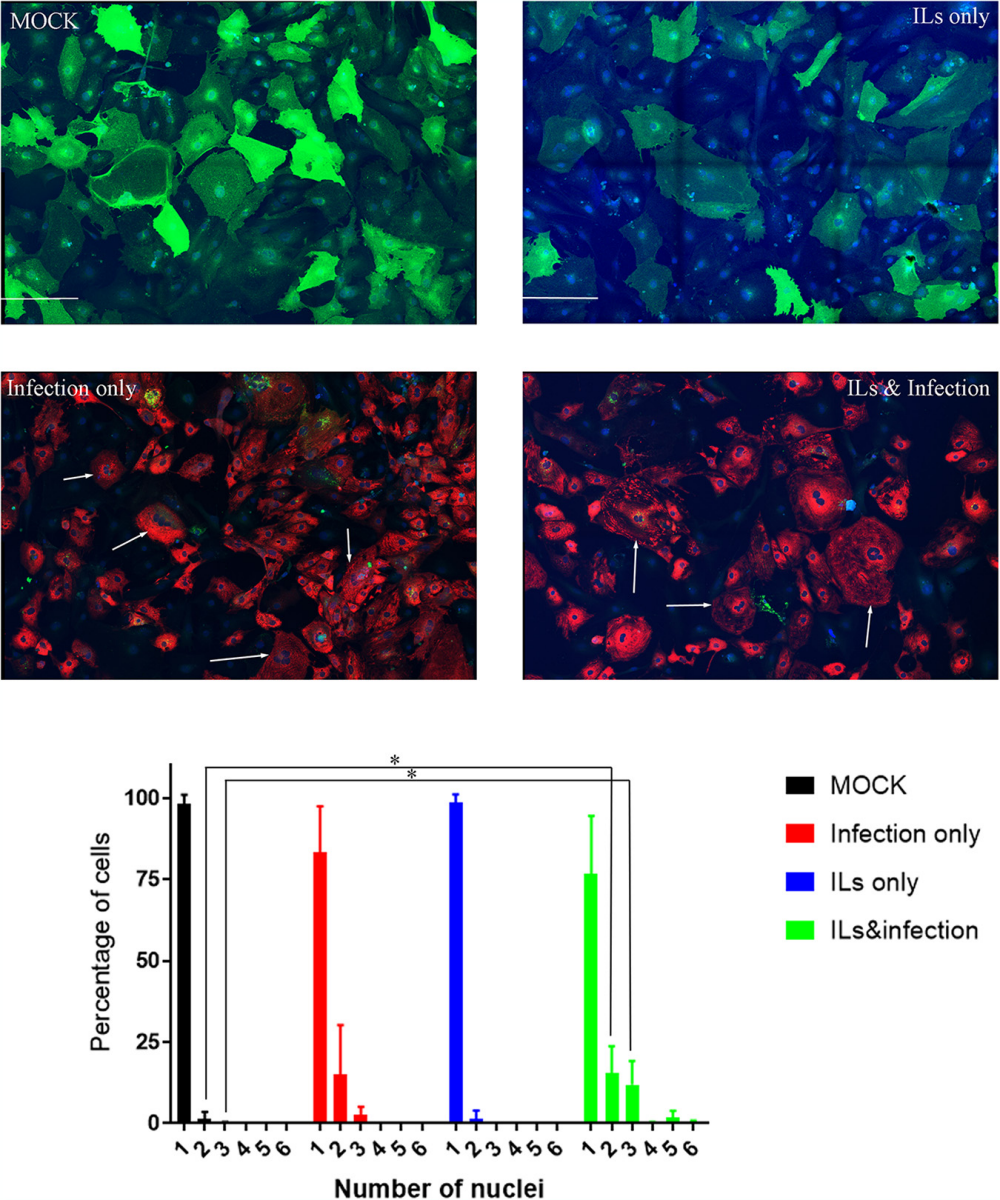
SARS-CoV-2 infection increases the percentage of multinucleated cells. Cells of CM line MSN-31-01S were treated with ILs and/or infected with SARS-CoV-2 at an MOI of 0.1 for 48 h. Shown is immunostaining of CMs for ACE2 (green), NP (red), and nuclear stain (blue). Mock-infected cells express ACE2 and predominantly have a single nucleus, but some double nuclei are detected. ILs did not alter the count of nuclei in these CM cells. Infection with SARS-CoV-2 at an MOI of 0.1 for 48 h does diminish the amount of ACE2 staining, and several cells are detected with 3 nuclei, as noted by the white arrows. Cells exposed to ILs and infection have an increase in the number of nuclei, where 3 to 5 nuclei are pointed out by the white arrows. The graph is the average number of nuclei counted between 3 independent experiments. Statistical significance was determined by unpaired *t* test, and a result was significant at *P* < 0.05 (*) comparing control to IL treatment and SARS-CoV-2 infection for 2 and 3 nuclei per cell. The scale bar in each image is 200 μm.

We observed over several CM lines that infection by SARS-CoV-2 can lead to the formation of multinucleated cells with 3 or more nuclei present in one cell (Fig. 5). Mock-infected cells expressing ACE2 (green) and nuclei (blue) predominantly have a single nucleus, with some noted double nuclei present. IL treatment does not alter the count of nuclei compared to mock infection in these cells. However, infection with SARS-CoV-2 (red) does increase the number of CM cells with 3 nuclei. The number of nuclei increases further when we have infection along with ILs, where we detect CM cells with 5 or 6 nuclei. Multinucleated cells generated during SARS-CoV-2 infection are likely to be the result of syncytium formation due to SARS-CoV-2 S-mediated fusion of plasma membranes of infected cells (31).

**FIG 6:**
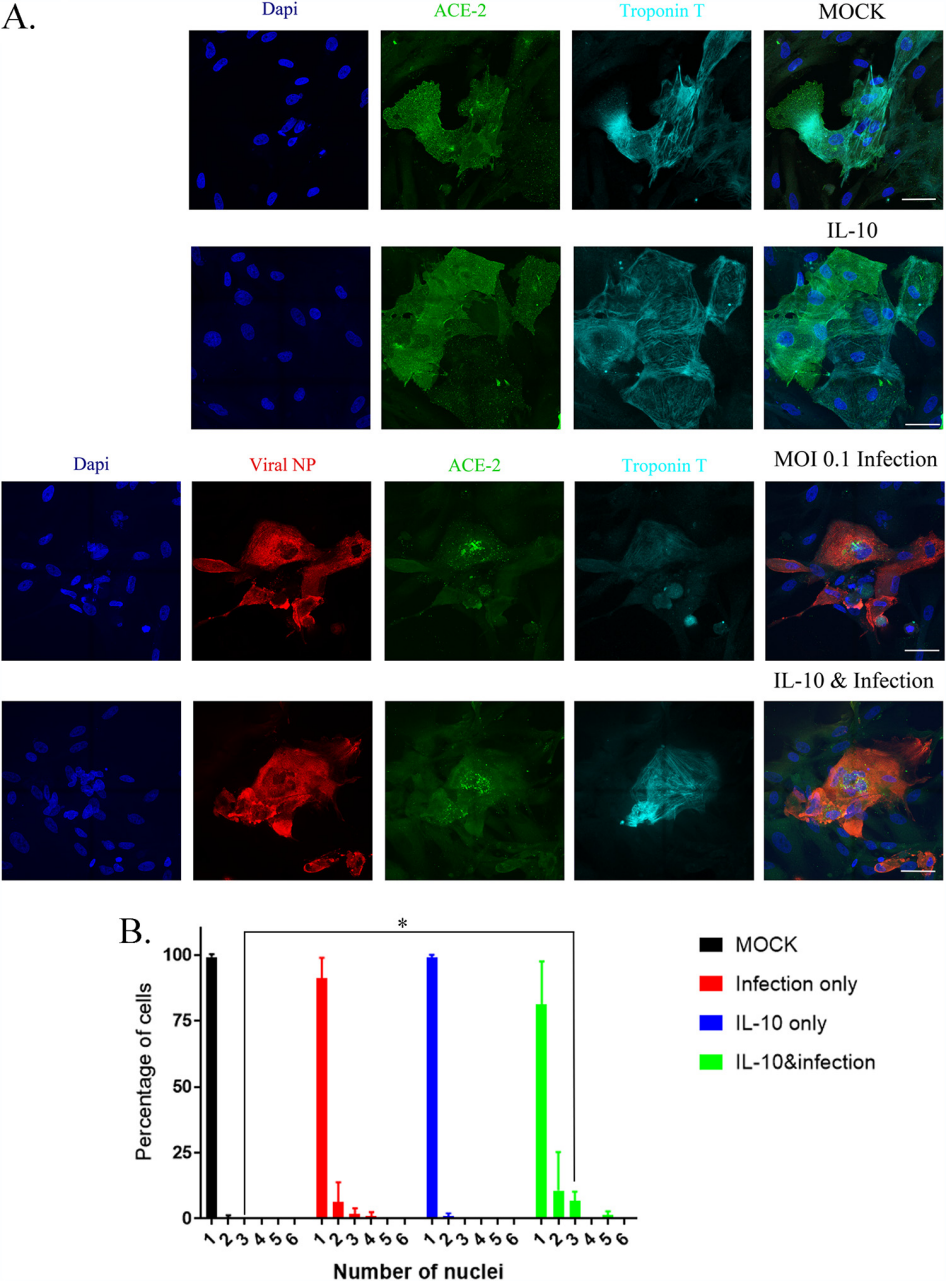
IL-10 does not increase cell diameter of CM cells, but it does increase the number of multinucleated cells. (A) We tested three different CM lines; results from CM line MSN31-01S are shown. We treated the cells with 30 ng/mL IL-10 and then infected them with SARS-CoV-2 at an MOI of 0.1 for 48 h. Mock-infected cells express both troponin T (cyan) and ACE2 (green), and cells treated with IL-10 alone look similar to mock-infected cells. CM cells infected with SARS-CoV-2 have decreased detectable ACE2 and are infected, as detected by viral NP antibody staining in red. However, there is an increase in the number of multinucleated cells with infection only. Combining IL-10 and SARS-CoV-2 infection, we do not see a detectable change in cell size, but there is a consistent decrease in ACE2 expression, as well as a visible increase in multinucleated cells. (B) The number of multinucleated cells for each treatment is tabulated in the graph, and the numbers are the average from three different CM lines (MSN31-01S, MSN07-07S, and MSN12-04S). Statistical significance was determined by unpaired *t* test, and a result was significant at *P* < 0.05 (*) comparing control to IL-10 and SARS-CoV-2 infection for 3 nuclei per cell.

**FIG 7:**
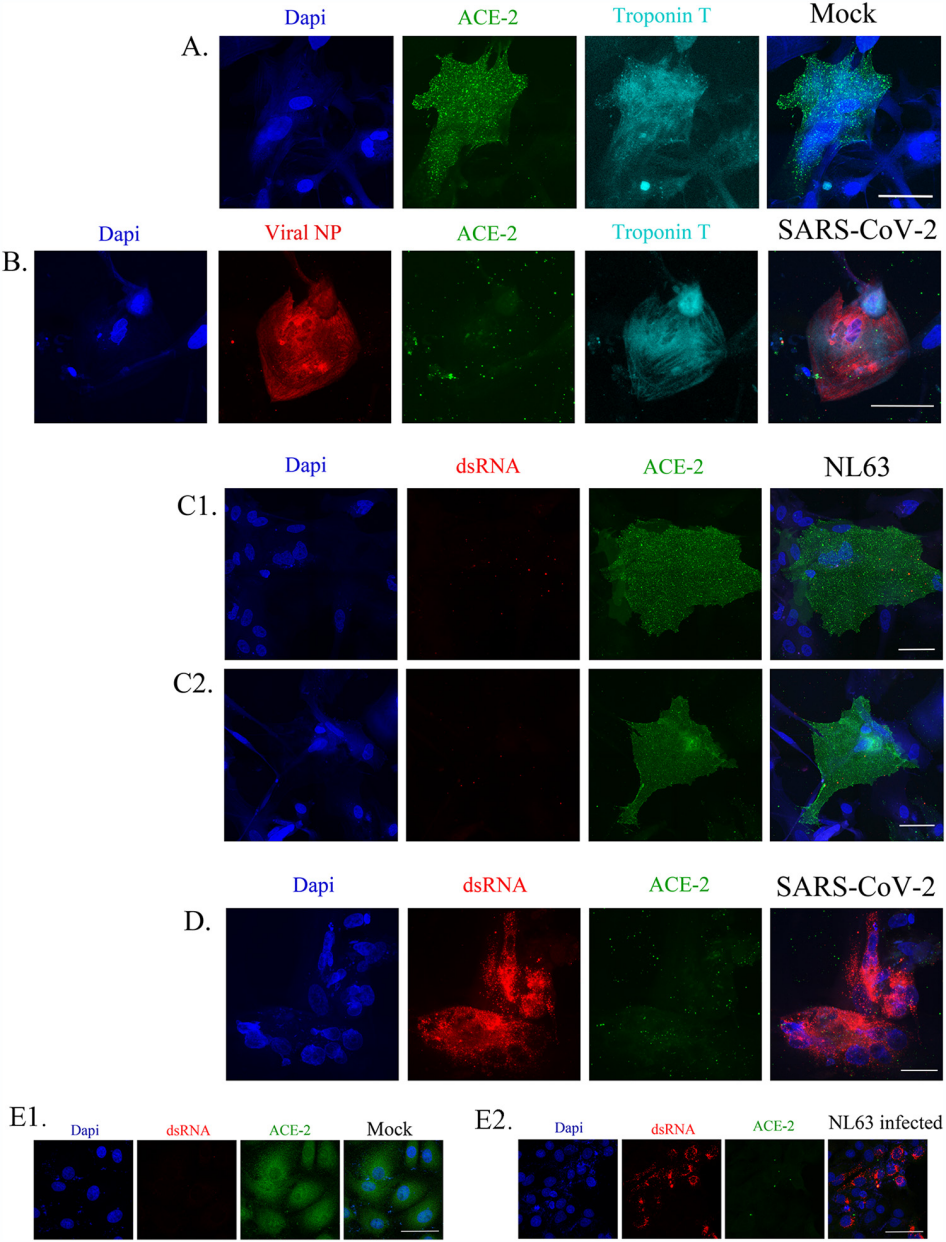
NL63, a seasonal coronavirus that can infect human cells, cannot infect cardiomyocyte cells. (A) We tested three different CM lines; results from CM line 12-4 are shown. CM cells do express ACE2 (green) and troponin T (cyan), as shown by the mock-infected cells. (B) CM cells can be infected with SARS-CoV-2 (red; viral NP antibody), as shown by the decrease in viral NP antibody and ACE2. (C1 and C2). In duplicate, we show NL63 infection at an MOI of 0.1 for 48 h. These cells express ACE2 but have no detectable infection when we look for dsRNA with an antibody (red). (D) The dsRNA antibody is working, as with SARS-CoV-2 infection, we see that this CM line is infected by dsRNA detection (red), and ACE2 levels are decreased in these cells. We also observe an increase in multinucleated cells (blue). (E) To confirm the lack of infection of NL63 in CM lines, we confirmed that the NL63 virus was active by infecting LLC-MK2 cells (E2), a cell type that expresses ACE2 and is known to be infected by coronaviruses. LLC-MK2 cells do express ACE2 (“Mock” in panel E1), and when we infect them with NL63 at an MOI of 0.1 (E2), ACE2 is diminished, and we detect dsRNA (red) by antibody staining. The scale bar is 50 μm.

We also used another interleukin, IL-10, in combination with SARS-CoV-2 and found there were no significant changes in cell diameter of the same CM lines with IL-10 alone or in combination with SARS-CoV-2 at an MOI of 0.1 (Fig. 6A and B). We also tested a coronavirus strain, HCoV-NL63 (NL63 [NR-470] from BEI Resources), known to infect humans using the ACE2 receptor and found at MOI 0.1 it could not infect 3 different CM lines (Fig. 7C, panels 1 and 2) (32). We confirmed that the NL63 virus was active by infecting LLC-MK2 cells, a cell type that expresses ACE2 and known to be infected by coronaviruses (Figure 7E, panels 1 and 2).

**FIG 8:**
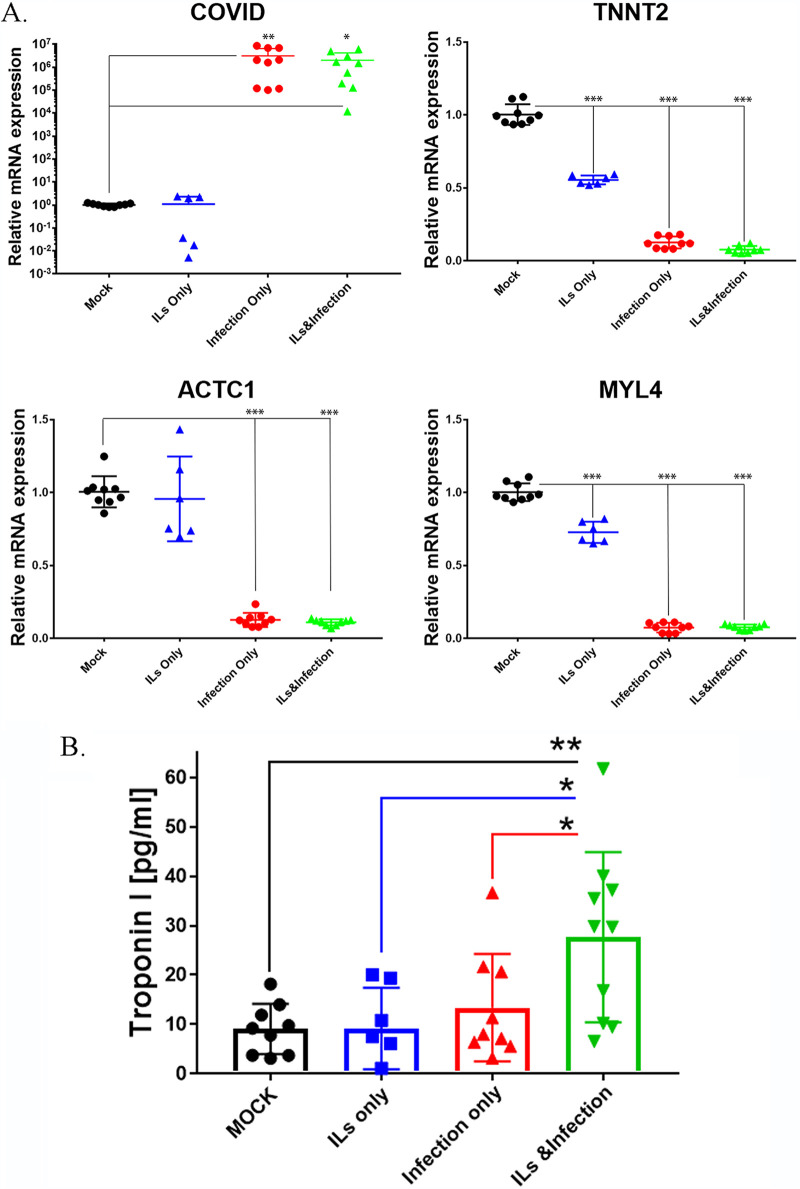
SARS-CoV-2 disrupts the contractile mechanism of CM lines tested, as determined by RT-qPCR. (A) Relative mRNA expression in human iPSC-derived cardiomyocytes obtained from three healthy subjects as determined via RT-qPCR. High levels of COVID-19 mRNA expression were detected in iPSC-derived CMs infected with COVID-19 virus only and with COVID-19 virus plus ILs, but not detectable in cells mock infected or treated with ILs only, confirming viral infections. Cardiac contractile genes, including those coding for troponin T (TNNT2), myosin light chain 4 (MYL4), and cardiac actin (ACTC1), showed a 90% ± 5% reduction in their expression in iPSC-derived CMs infected with COVID-19 virus only and with COVID-19 virus plus ILs compared to mock infected. These levels of contractile gene expression showed a 25% ± 10% reduction when iPSC-derived CMs were treated with IL only. These findings correlated with iPSC-derived CMs’ contractility. Statistical significance was determined by one-way ANOVA with Bonferroni multiple-comparison test: *, *P* < 0.05; **, *P* < 0.01; ***, *P* < 0.001. (B) Three different CM lines were treated with ILs and/or infected with SARS-CoV-2 at an MOI of 0.1 for 72 h. The supernatants were collected and used for the measurement of levels of troponin I released into the medium. We found cells treated with ILs or infected with SARS-CoV-2 did not significantly release troponin I into the medium. However, infection in the presence of ILs significantly increased release of troponin into the culture medium. Comparing IL treatment or infection only to the combination of ILs with infection, we see there was significant increase in troponin release when we combined ILs with infection. Statistical significance was determined by one-way ANOVA with Bonferroni multiple-comparison test: *, *P* < 0.05; **, *P* < 0.01.

Since the myofibrils are disorganized after infection, we determined the effects of infection on the contractile apparatus CM lines in the absence or presence of IL-6 and IL-1β. For this, we used reverse transcription-PCR (RT-PCR) to measure levels of cardiac troponin, actin, and myosin light chain (Fig. 8A). Probing for SARS-CoV2, we see significant increases with infection of SARS-CoV-2, which is similar to the results from combination with IL-6 and IL-1β (ILs). In the absence of the virus, we had no detectable levels of SARS-CoV-2 in mock-infected or IL-treated cells alone. We looked at mRNA levels of cardiac contractile proteins. Expression of cardiac troponin T (TNNT2), an important structural component of cardiomyocytes, was strongly downregulated in cells infected with SARS-CoV-2. IL-6 and IL-1β treatment also significantly reduced expression of troponin T (by nearly 50%), and this was greatly enhanced by infection with SARS-CoV-2. We also measured levels of t cardiac actin (ACTC1) and myosin light chain (MYL4) mRNAs. SARS-CoV-2 strongly repressed expression of both ACTC1 and MYL4. ILs alone did significantly reduce levels of MYL4 expression, but in the combination with the virus, there was strong level of repression of both ACTC1 and MYL4.

An important indicator of CM damage is the release of troponin from the cells. Hence, we measured the levels of troponin I in the culture medium of cells without and with infection and in the absence and presence of interleukins. For this, we used a commercial enzyme-linked immunosorbent assay (ELISA) kit and measured culture medium troponin levels from three different CM cell lines (Fig. 8B). IL treatment did not cause the release of troponin. However, when CMs were infected with SARS-CoV-2 at an MOI of 0.1, there was increased release of troponin into the culture medium, but the effects were not statistically significant. Importantly, under these conditions, infection in the presence of ILs caused a statistically significant near-3-fold increase in levels of troponin I in culture medium (Fig. 8B). These results indicate that infection in the presence of interleukins can substantially damage cardiomyocytes, although interleukins alone or infection in the absence of interleukins can also damage cardiomyocytes. *In vivo*, this distinction may not be relevant as infection invariably increases interleukin levels in blood.

### Effects of SARS-CoV-2 infection on beating of ventricular cardiomyocytes and left ventricular ejection fraction show similar characteristics.

**FIG 9:**
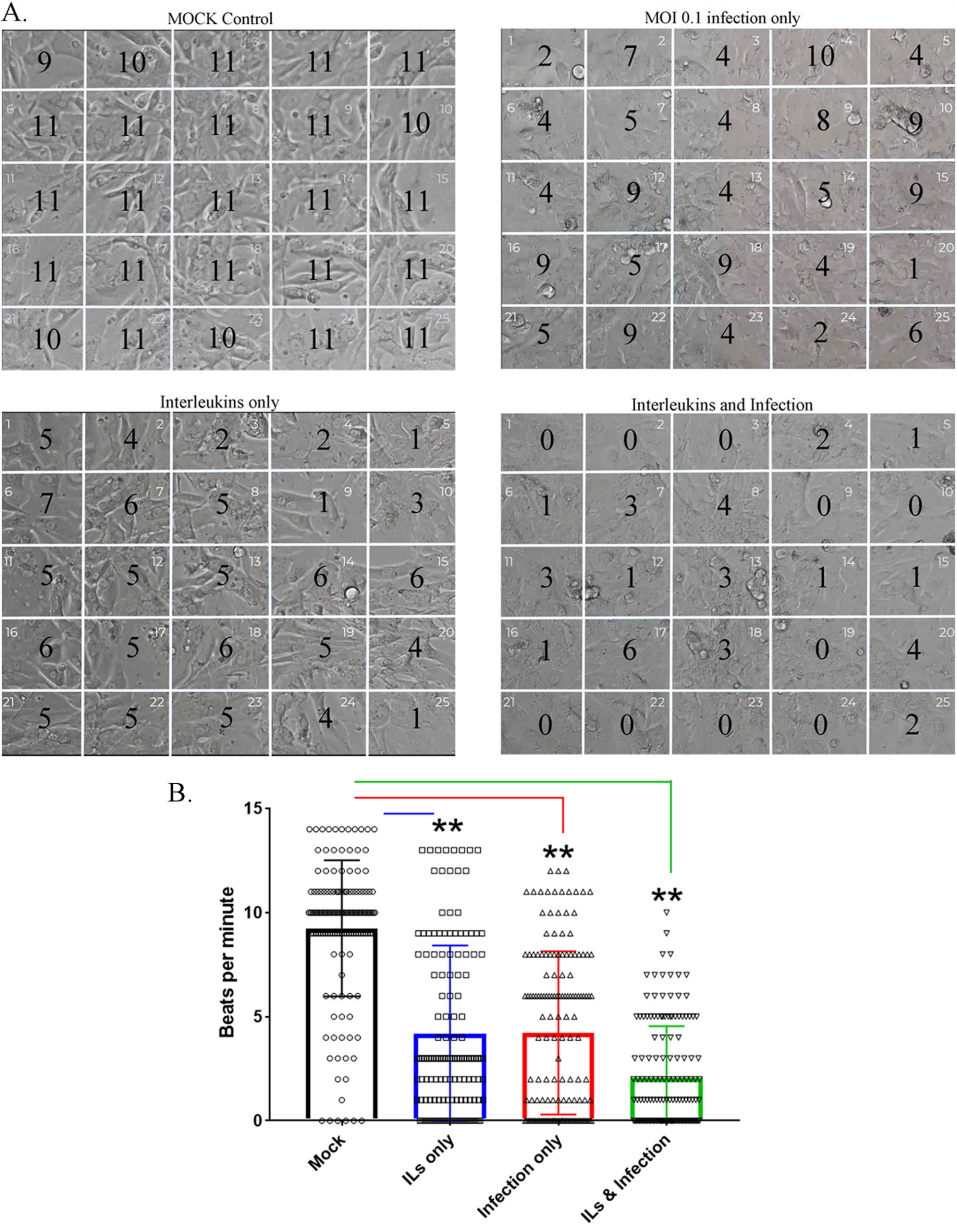
Effect of SARS-CoV-2 infection on beating of ventricular cardiomyocytes in cultures. (A) Three different CM lines were recorded for their beating in culture with ILs and/or infection in a biosafety level 3 facility. These CM lines were cultured for 45 to 60 days. Onto the movies that we recorded, we juxtaposed a 5-by-5 grid to count the number of beats per min for each cell line at 48 h postinfection. The number in each of the 25 grids (beats per minute) is the average from two experiments for one representative CM line for each condition. We observed that infection alone can slow down the beating, as could IL treatment. The strongest attenuation of beating was observed with SARS-CoV-2 infection in the presence of ILs. These images are from CM line MSN08-06S. Movie S1 (https://iyengarlab.org/) shows the beating of the CMs under various conditions. (B) Summaries of the counts of the beating in three different 3 CM lines are shown. Beat measurement in each area of the grid is represented as an open dot, and the mean and standard error of the mean (SEM) are shown. Asterisks indicate significance at *P* < 0.001 by ANOVA with Bonferroni multiple-comparison test. Though all conditions significantly attenuated beating, the most robust effect was from combining ILs with infection with SARS-CoV-2.

A well-known hallmark for healthy CMs derived from iPSC lines is their ability to beat in a coordinated manner *in vitro* (33). We recorded the beating of 3 different CM lines under the following conditions: mock infection, infection with SARS-CoV-2 at an MOI of 0.1, treatment with ILs alone, and infection in the presence of ILs 48 h postinfection (Fig. 9A; see related Movie S1 at https://iyengarlab.org). To quantitate the number of beats per minute, we counted how many beats were occurring over 1 min within 5-by-5 grids (25 grids in total) superimposed over the video. Counting was done by researchers who were blind to treatment conditions. In Fig. 9A, we show a representative snapshot of the field of recording with the number of beats per minute. Control cells show coordinated beating over the entire field (Fig. 9A, upper left panel). Infection with SARS-CoV-2 at an MOI of 0.1 causes considerable alteration in coordinated beating such that the beating is decreased in some regions, while it is near normal in several regions (Fig. 9A, right panel). Treatment with interleukins alone causes similar effects, although in all regions we see a decrease in beat frequency (Fig. 9A, lower left panel). Infection in the presence of interleukins results in a marked decrease in beating, with no beats in 9 of the 25 fields (Fig. 9A, bottom right panels). Summary statistics across the multiple cell lines show that both interleukins and infection decrease beating (4.17 and 4.22 beats/min, respectively, versus 9.3 beats/min for the control), with a more pronounced decrease (2.08 beats per min) when infection occurs in the presence of interleukins, which is likely to be the most physiologically relevant scenario (Fig. 9B).

**FIG 10:**
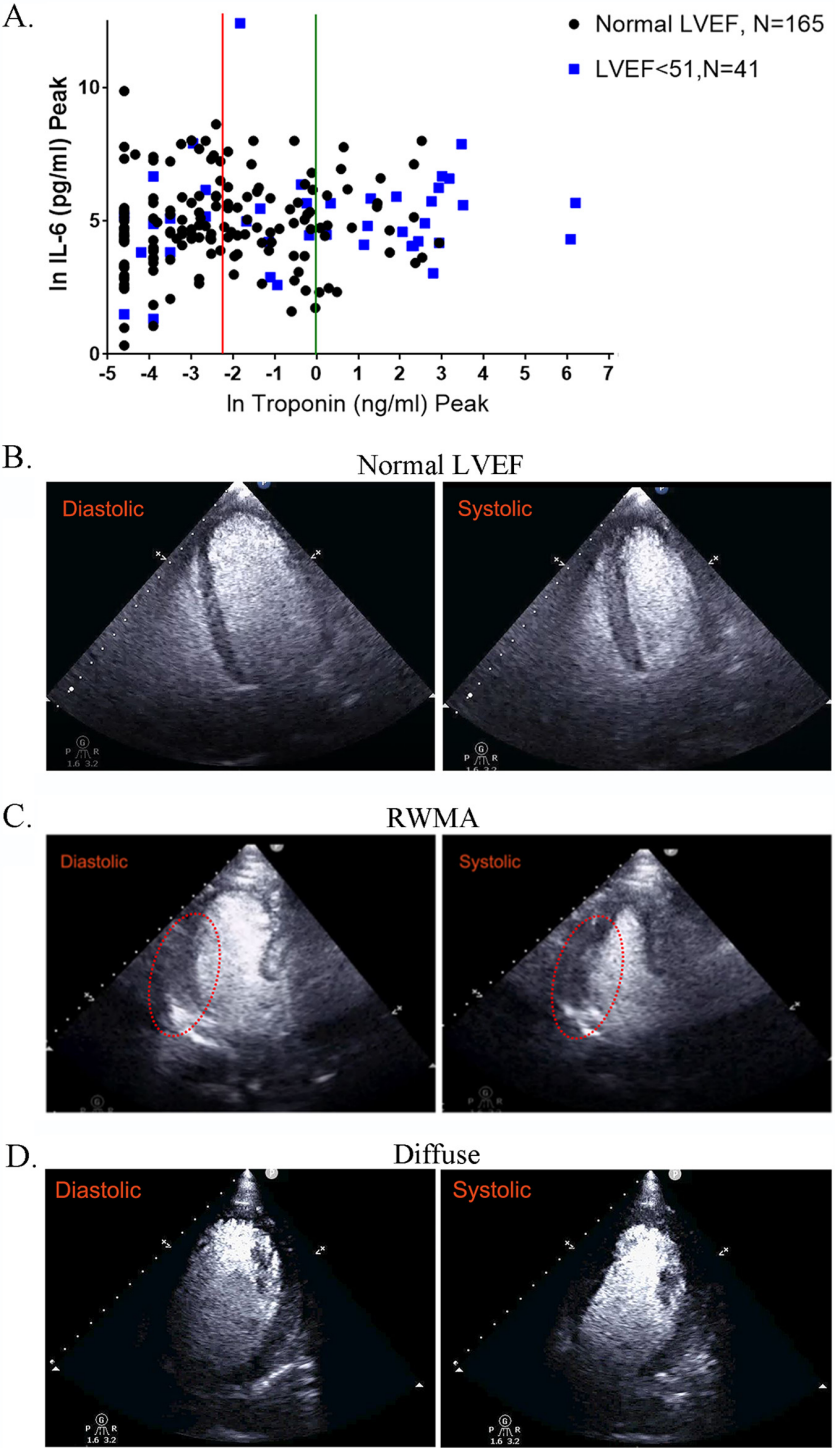
Effect of SARS-CoV-2 infection on cardiac output in COVID-19 patients. (A) COVID-19 patients without prior cardiac disease were divided into two groups: patients with normal LVEF (*n* = 165) and those with a decreased LVEF of <51 (*n* = 41). We plotted the peak troponin I level (ng/mL) using a natural log scale (ln) and peak IL-6 (pg/mL). The red line indicates where troponin levels are at 0.1 ng/mL (ln = −2.3) and the green line indicates levels at 1 ng/mL (ln = 0). For the group with LVEF of <51, 20 out of 41 (50%) have troponin levels of >1 ng/mL, while the number with normal LVEF is 22/165 (13.3%). In another group of patients with LVEF of <51, 6/41 (14.6%) have troponin I levels greater than 20 ng/mL (ln = 3); none of the patients with normal LVEF have a troponin I level above 20 ng/mL. For troponin I, the difference is very significant by *t* test (*P* < 0.0001), when comparing the group with normal LVEF to the group with LVEF of <51. (B to D) Echocardiogram clips showing diastolic and systolic states. (B) “Normal” demonstrates an apical 4-chamber view (after administration of an ultrasonic enhancing agent) from a transthoracic echocardiogram obtained from a COVID-19 patient (see Movie S2 at https://iyengarlab.org). The findings are consistent with preserved left ventricular ejection fraction and no regional wall motion abnormalities (diastolic and systolic frames). The end-diastolic and end-systolic volumes are 104 mL and 35 mL, respectively, with a left ventricular ejection fraction of 66%. (C) Regional wall motion abnormality (RWMA) demonstrates an apical 3-chamber view (after administration of an ultrasonic enhancing agent) from a transthoracic echocardiogram obtained from a COVID-19 patient (see Movie S3 at https://iyengarlab.org). The findings are consistent with basal and mid infero-lateral wall hypokinesis, despite preserved left ventricular ejection fraction (diastolic and systolic frames). The RWMA is highlighted by the red oval. The end-diastolic and end-systolic volumes are 102 mL and 50 mL, respectively, with a left ventricular ejection fraction of 51%. (D) “Diffuse” demonstrates an apical 4-chamber view (after administration of an ultrasonic enhancing agent) from a transthoracic echocardiogram obtained from a COVID-19 patient (see Movie S4 at https://iyengarlab.org). The findings are consistent with diffuse left ventricular wall hypokinesis and mildly decreased left ventricular ejection fraction. The end-diastolic and end-systolic volumes are 160 mL and 90 mL, respectively, with a left ventricular ejection fraction of 44%.

Since the beating of CMs in culture is broadly indicative of myocyte contraction *in vivo*, we reasoned that alterations in contractility during COVID-19 in patients could result in a decrease in left ventricular ejection fraction (LVEF), a physiological measure routinely used in clinical diagnosis. Hence, we looked for COVID-19 patients without prior cardiac disease who had elevated troponin levels and showed decreased LVEF. Using data from two Mount Sinai Health System hospitals, we identified a cohort of 206 COVID-19 patients without indications of prior heart disease for whom imaging data along with troponin I and IL-6 levels were available. Patient data for peak troponin and IL-6 levels for such patients are shown in Table 1. In Fig. 10A, we show a plot of COVID-19 patients with normal LVEF (black dots) and reduced LVEF (blue squares) in terms of their relationship to blood IL-6 and troponin I levels. We find that nearly 50% (20/41) of the patients with an LVEF of <51 had troponin levels above 1, while for normal LVEF, it is 13.3% (22/165). We also find that 6/41 (14.6%) patients have troponin I levels greater than 20 ng/mL (ln of 3), and none of the normal LVEF patients are above 20. No relationships between IL levels and LVEF status could be readily observed. Representative cardiac images from COVID-19 patients are shown in Fig. 10B to D. These are apical views after administration of an ultrasonic enhancing agent for transthoracic echocardiogram (TTE). A COVID-19 patient with no known cardiac disease and normal LV function is shown (Fig. 10B; see related Movie S2 at https://iyengarlab.org), where no detected abnormalities are found in the diastolic and systolic frames. Clips of the diastolic and systolic frames are shown in Fig. 10B. The end-diastolic volume (EDV) and end-systolic volume (ESV) are 104 and 35 mL, respectively, with a left ventricular ejection fraction of 66%. Two patients with significantly elevated levels of blood troponin and reduced LV function (LVEF, <51%) are shown in clips in Fig. 10C and D. One patient had a mid infero-lateral wall hypokinesis, but preserved LV ejection fraction (Fig. 10C; see related Movie S3 at https://iyengarlab.org/). The regional wall motion abnormality (RWMA) is pointed out in the red oval in Fig. 10C. The EDV and ESV are 102 and 50 mL, respectively, with an LVEF of 51%. The second patient with newly reduced LV function had global LV dysfunction. From the clips in Fig. 10D (see related Movie S4 at https://iyengarlab.org), the findings are consistent with diffuse LV wall hypokinesis and mildly decreased LVEF, and the EDV and ESV are 160 and 90 mL, respectively, with LVEF of 44%. Overall, the relationship between elevated troponin I level and decreased LVEF is highly significant in COVID-19 patients.

**TABLE 1:** Patient data for peak troponin and IL-6 levels recorded for COVID-19 patients with no known cardiac dysfunction^*a*^

Parameter	Result for patients COVID-19 positive and with no known history of cardiac disease^*a*^		Significance by *t* test^*b*^
	Normal LVEF	Newly reduced LVEF	
No. of patient samples	165	41	
Peak IL-6, pg/mL	111.5 (IQR, 49–296)	146 (IQR, 63.55–355.6)	NS
Peak troponin, ng/mL	0.062 (IQR, 0.02–0.375)	1.27 (IQR, 0.07–14.53)	*P* < 0.0001

a Patients are broken into two groups: one with normal left ventricular ejection fraction (LVEF) and the other with newly reduced LVEF (LVEF, <51). The data are presented as the median value with IQR in parentheses.

b There was no significant difference (NS) between the two groups for peak IL-6, but there was a very significant difference in troponin (*P* < 0.0001).

## DISCUSSION

As a respiratory virus, the multiorgan pathophysiology of SARS-CoV-2 could not entirely be predicted. Although the primary pathology is in the lung, the effects of SARS-CoV-2 infection on other organs cannot be overlooked. Post-acute COVID-19 syndrome (34) is now well recognized and affects many organs, including the heart, kidney, and nervous system. Although it has been long known that ACE2, the receptor for members of the SARS family, is widely distributed (10, 35), extensive infection of the virus in organs other than the lungs has not been observed. Nevertheless, several groups have shown the sparse presence of virus in heart tissue of COVID-19 patients or detected the mRNA of SARS-CoV-2 by RT-PCR in endomyocardial biopsy specimens (36–41).

Analyses of the clinical data from the hospitals of the Mount Sinai Health System indicates that significant numbers (>10%) of COVID-19 patients develop cardiac dysfunction during infection. If these ratios hold worldwide, then nearly 16 million individuals would have the potential for cardiac dysfunction that could lead to long-term heart disease. The data from long-term studies of 9/11 responders indicate that this is likely to be the case (42, 43). Hence, understanding the origins of cardiac dysfunction in COVID-19 patients will be important for clinical management and therapeutic strategies to mitigate the development of heart disease when treating patients at centers for post-COVID care, such as the one in the Mount Sinai Health System.

Myocardial damage can occur due to multiple reasons, including decrease in oxygen levels due to acute respiratory distress syndrome (ARDS), the formation of microthrombi, as has been observed in COVID-19 patients (15), and direct injury of cardiomyocytes by viral infection and the inflammatory state of the COVID-19 patients. Our *in vitro* cardiomyocyte studies indicate that direct injury due to infection is likely to be one of the causes of myocardial injury in COVID-19 patients. Two aspects of our *in vitro* studies are noteworthy: first, measurable cardiomyocyte injury, such as release of troponin into the culture medium and near-complete disruption of coordination and inhibition of beating, is greatest when viral infection occurs in the presence of interleukins, a scenario that is most likely similar to that seen *in vivo*; second, interleukins do not increase the number of cells infected which remains stable around 35%. Clinical data have suggested that myocardial inflammation might persist for more than 2 months after infection (44), although the general applicability of these findings need to be established. These observations suggest that effects of COVID-19 on cardiac contractility could be long lasting and could result in heart failure (45). A third aspect of our *in vitro* studies elucidated the hypertrophy of cardiomyocytes by cytokines. Cardiac hypertrophy can occur in response to proinflammatory cytokines, such as IL-6 and IL-1β, and can be required for maintenance of cardiac homeostasis (46). Cardiomyocytes are terminally differentiated and demonstrate plasticity in response to the alterations in their environment—both physiological and pathological ones. Hypertrophy enables cardiomyocytes to enlarge their capillary network to receive adequate nourishment under normal physiological conditions. However, in a pathological setting, this hypertrophy could exceed the capacity of capillaries to adequately supply nutrients (46). Patients infected with SARS-CoV-2 could have cardiac hypertrophy both in response to the lung infection and diminished oxygen supply or due to direct infection of cardiac tissues. Further studies are required to better understand this complex hypertrophy of cardiomyocytes.

Taken together, our clinical and cell-level observations suggest that cell communication between cardiomyocytes and other cell biological mechanisms can underlie the effects of SARS-CoV-2 infection in myocardial tissue. Such potential mechanisms merit detailed studies under conditions that consider genomic determinants that could control the pathophysiological responses both acute and long term.

In conclusion by integrating analyses of clinical data and observations from cell-level experiments in culture, using qualitative matches between biomarkers like troponin and physiological functions like beating, we show that direct infection of cardiomyocytes can be one mechanism of myocardial injury during COVID-19.

## MATERIALS AND METHODS

### Study approval.

This study was approved by the Institutional Review Board (IRB) of the Icahn School of Medicine at Mount Sinai (study 20-0156, Myocardial injury in COVID-19) and complies with all institutional and federal requirements regarding research using data from human subjects.

### Clinical data and study population.

This retrospective analysis was conducted using deidentified electronic medical record (EMR) data from patients diagnosed as COVID-19 positive within the Mount Sinai Hospital System between 9 March and 15 July 2020. The Mount Sinai Health System is a network of 8 hospital campuses and over 400 ambulatory practices spanning the New York metropolitan area (17). COVID-19 diagnosis was based on positive-PCR-based clinical laboratory testing for the SARS-CoV-2 virus. Patient’s data at Mount Sinai Health System were collected by Mount Sinai Data Warehouse team, and deidentified data were released for research purposes.

All collected data and events occurring during the time that medical attention was provided to the patient during the visit were defined as encounter data. For patients with more than one documented encounter in the data set, such as those with a readmission or transfer from another affiliated facility, only the first encounter data were included for analysis. Patient characteristics were collected from the EMR. Basic demographics included age, sex, race, ethnicity, body mass index (BMI), and smoking status (current smoker, former smoker, never smoker, or passive smoker). The patient’s vitals at admission (temperature maximum, systolic blood pressure minimum, oxygen saturation minimum, heart rate, etc.), their maximum/minimum values recorded during their stay, and comorbidities present on admission (e.g., cancer, hypertension, heart failure, cardiac arrhythmias, cerebral infarction, chronic kidney disease, acute kidney injury, HIV/AIDS, liver disease, diabetes, etc.) were also collected from the EMR. Lab data of patients were collected several times during the encounter, and the maximum value recorded was collected for all lab features, except a few for which minimum values were recorded (albumin, eGFR, hemoglobin, percentage of neutrophils, hematocrit, platelet count, and ferritin) based on severity.

Under an agreement with the institutional review board (IRB), which is a precursor to and separate from our study, the Mount Sinai Data Warehouse released deidentified clinical data of all patients with COVID-19 who had or were undergoing treatment in the Mount Sinai Health System. Since deidentified data were used, patient consent was not sought. We did univariate significance analyses of the differences in continuous features between patients without cardiac disease and patients with cardiac disease using Student's *t* test and of categorical features using the χ^2^ test in all the resultant data sets.

Raw data from 9 March to 15 July were acquired from the Mount Sinai Data Warehouse (MSDW) and underwent the preprocessing steps described below.

### Cardiac imaging data.

Cardiac point-of-care ultrasounds/echocardiograms were performed by certified emergency room physicians and critical care physicians on patients when deemed necessary by the attending physician. Formal echocardiograms were done on those for whom further information was necessary. The patients were identified retrospectively, and subsequent data collection occurred to obtain baseline characteristics, prior medical history, including cardiovascular disease history, hospital outcomes of mortality and intubation, anthropometric measurements, and laboratory values.

### Analyses of MSDW data. (i) Data preprocessing.

We applied the basic preprocessing steps listed below to the raw clinical data in the development set.

1. Only records for patients with a positive SARS-CoV-2 test were retained, and the rest were eliminated.
2. Only the first record of encounter data among all those with the same patient ID was retained, and the rest were eliminated.
3. Vital sign minimum and maximum values (e.g., systolic blood pressure minimum and systolic blood pressure maximum) were retained during the course of the encounter.
4. Lab data maximum values were retained during the encounter, except for a few features for which minimum values were calculated (albumin, eGFR, hemoglobin, percentage of neutrophils, hematocrit, platelet count, and ferritin).
5. Merged data (encounter data, vital signs data, and lab data) were included on the patient ID.
6. Features with missing values in more than 60% of the patient records (seven in this case) were dropped to prevent their adverse effects on data analyses.
7. Any of the categorical features (e.g., sex and race) that had exactly the same value for over 99% of the patients (i.e., were effectively constant) were removed, since they were unlikely to be predictive. The values of the remaining categorical features were converted into numerical form (1, 2, 3…) based on the order of the discrete values appearing in the data. Undefined or unclear entries for these features were recorded as missing values.
8. The continuous features (e.g., age and O_2_ saturation minimum) were normalized by converting them into respective Z-scores by applying the formula (*X* − μ)/σ, where *X*, μ, and σ are the value, mean, and standard deviation of a given feature, respectively.

The same preprocessing steps were applied to the test set. In the steps where the preprocessed values of the features were dependent on those determined from the development set (e.g., numerical values of categorical features in step 7 and mean and standard deviation in step 8), the same values determined from the development set were applied to the test set. This was necessary to ensure that a predictor trained on the development set is applicable to the test set. It also had the desirable effect of the steps not being dependent on the relatively smaller sizes of the test set, which may make it difficult to estimate parameters like mean and standard deviation in step 8 reliably.

The resulting development and test set were used for the machine learning analyses described in the following sections.

### (ii) Development and evaluation of predictive machine learning models.

For predictor construction, we further randomly split the development set into training and holdout subsets of sizes 75% (*n* = 2,536) and 25% (*n* = 846), respectively. The analysis steps used in this process, specifically missing value imputation and feature selection (described below), were used to learn candidate predictors from the training set, which were in turn evaluated on the corresponding holdout set. The logistic regression (LR), as implemented with its default parameters in Python’s scikit-learn library, and the eXtreme Gradient Boosting (XGBoost) algorithm (47), as implemented with its default parameters in Python’s XGBoost package, were used to develop these candidate predictors. This process was repeated a hundred times. The performance of the candidate predictors was quantified as the average value of their evaluation measure in each round. The overall predictor development process was followed as shown in Fig. 2A.

All predictor development and validation were conducted using the following parameters: area under the receiver operating characteristic (ROC) curve (AUC score) (48), area under the precision recall curve (AUPRC), precision maximum (P_max), recall maximum (R_max), F_max (i.e., the f1 score, which is the harmonic mean of precision and recall), and accuracy as the evaluation metric.

### (iii) Missing value imputation.

Several features in our data set had missing values (Table S2). To maximize feature inclusion in the predictive modeling process, we evaluated the impact of imputing and including missing values among features (48). Specifically, we considered an increasing number of features with increasing levels of missing values (≤0%, 5%, 10% … 60%) in the training subset of the development set. Then, we replaced the missing values for each feature in the development set with its mean or mode in the training subset, depending on whether the feature was continuous or discrete, respectively. Finally, we trained and evaluated candidate predictors using the two algorithms listed above on the training and holdout subsets of the development set, respectively. Using the results, we estimated the level of missing values that would be the most beneficial to be included and imputed.

### (iv) Feature selection.

In predictive modeling with clinical data, several features may be irrelevant to the target problem, and several may be redundant with other features, making accurate prediction challenging (49). To address this challenge and to develop a predictor more parsimonious than one based on all features, we utilized established feature selection methods (49). Specifically, we adopted the same training-holdout setup and classification algorithms described in the previous subsection and assessed how well features were selected automatically by using the Recursive Feature Elimination (RFE) algorithm (50). This algorithm ranks the full set of features and then evaluates the performance of a classifier trained using one of our two candidate algorithms on the *k* top-ranked features, where values of *k* are prespecified. Based on the evaluation results, RFE infers the value of *k* and as many features that are likely to perform the best with the given algorithm. The candidate values of *k* we tested within RFE were 1, 5, 10, 15, 20, 25, 30, 40, 50, 60, 70, and 86. For both the classification algorithm and RFE-inferred value of *k*, we selected the final set of features as the top-*k* highest-frequency features selected by RFE for that algorithm across the 100 runs of the training-holdout setup.

### (v) Final predictor development and validation.

Once we had determined the acceptable level (% of patients) of missing values and the features satisfying this requirement, we imputed the corresponding missing values in the test set using mean or median values in the development set. Next, we trained and evaluated the final COVID-19 mortality predictor on the imputed development and validation sets, respectively. The predictor was trained using the both classification algorithms (XGBoost and logistic regression) on the top 15 selected features and the associated classification algorithm determined as described in the section “Previous selection.” The purpose of evaluating both of these predictors was to assess how their performances generalized to an independent patient population.

### (vi) Software.

The following Python version and packages were used in model building: Python 3.6.7, Pandas 0.24.2, NumPy 1.16.4, Scikit-learn 0.21.2, Scipy 1.3.1, Matplotlib 3.1.0, seaborn 0.9.0, xgboost 0.80, and MATLAB 9.5.0.944444 (R2018b).

### Biosafety.

All experiments involving SARS-CoV-2 were conducted under biosafety level 3 (BSL3) containment in accordance with the biosafety protocols developed by the Icahn School of Medicine at Mount Sinai. All experiments involving NL63 coronavirus were conducted under BSL2 containment in accordance with the biosafety protocols developed by the Icahn School of Medicine at Mount Sinai.

### Cells and viruses.

The SARS-CoV-2 USA-WA1/2020 strain, isolated from an oropharyngeal swab from a patient with a respiratory illness who developed clinical disease (COVID-19) in January 2020 in Washington, USA, was obtained from BEI Resources (NR-52281). The virus was propagated in Cercopithecus aethiops kidney epithelial cells (Vero E6 cells; ATCC, CRL-1586). Viral stocks were grown in Vero E6 cells as previously described and were validated by genome sequencing (51). We obtained the NL63 strain (NR-470) from BEI Resources. NL63 was isolated in 2003 from nasopharyngeal aspirate of human infant with acute respiratory disease in Amsterdam, The Netherlands. We amplified the virus in Macaca mulatta kidney epithelial cells (LLC-MK2; ATCC CCL-7.1) with Dulbecco’s modified Eagle’s medium (DMEM) supplemented with 2 mM l-glutamine, 1 mM sodium pyruvate, and 2% FBS. Both LLC-MK2 and Vero E6 cells were maintained in DMEM (Corning) supplemented with 10% FBS (Peak Serum) and penicillin-streptomycin (Corning) at 37°C and 5% CO_2_. All cell lines used in this study were regularly screened for mycoplasma contamination using the Universal Mycoplasma Detection kit (ATCC, 30-1012K).

### Healthy human subject iPSC-derived cardiomyocytes.

The induced pluripotent stem cell (iPSC) lines used in this study were obtained from a library of 40 lines from healthy human subjects (25). Five iPSCs (MSN02-04S, MSN07-07S, MSN08-06S, MSN31-01S, and MSN39-04S) were maintained in StemFlex medium (Gibco) on hES-qualified Matrigel (Corning)-coated 6-well cell culture plates at 37°C with 5% CO_2_. hiPSCs were passaged every 3 days and supplemented with Rock Inhibitor Y-27632 (SelleckChem) for 24 h after each passaging. For cardiomyocyte differentiation (52), hiPSCs were treated with 10 μM GSK3 inhibitor CHIR99021 (SelleckChem) in RPMI 1640 (Gibco) supplemented with B27 without insulin (Gibco) for 24 h. After an additional 48 h, medium was changed and then supplemented with 5 μM IWR1 (Sigma) for 48 h. From day 7 on, cells were cultured in RPMI 1640 medium supplemented with B27 with insulin (Gibco), and medium was changed every 3 days. Beating was generally observed around days 8 to 10. Between days 15 and 23, iPSC-derived cardiomyocytes were washed with Dulbecco’s phosphate-buffered saline (DPBS; Gibco) and cultured in RPMI 1640 without glucose and supplemented with 4 mM sodium lactate (Sigma) and 25 mM HEPES (Gibco) for metabolic switch purification (53). Lactate medium was refreshed every 4 days. After day 30, iPSC-derived cardiomyocytes were replated into assay plates for use in experiments. Typically, the cells were maintained for another 15 to 30 days before being subjected to treatment. Culture medium was changed every 3 days.

We also purchased from the Coriell Institute for Medical Research human iPSC-derived CMs labeled “Actin2 EGFR D60 atrial and ventricular.”

Infection of human cardiomyocytes was carried out at an MOI of 0.1 unless indicated otherwise. All experiments involving live virus were carried out under BSL3 containment. After the experiments, the virus was inactivated and cells fixed before the plates were removed from BSL3 containment for staining and further analyses.

All live cell beating recordings were made on an EVOS M5000 (Thermo) in the BSL3 containment facility. The cell lines used for these physiological experiments had been in culture for 45 to 60 days. Movies were made for 1-min recordings for 3 to 4 different fields per condition. These movies had a 5- by-5 grid (25 boxes) juxtaposed over the video image. We counted blind the number of beating cells per box for all conditions.

### Immunohistochemistry.

All microscopy was performed on a Zeiss LSM 880 confocal microscope with either a 20× or 63× oil lens. All cells were plated on 12- or 24-well glass-plated Mat-Tek dishes. For immunohistochemistry, all cells were permeabilized with 0.2% Triton-X and then blocked with either goat or donkey normal serum (Jackson ImmunoLabs). We used the following antibodies: anti-double-stranded RNA (anti-dsRNA) antibody (clone rJ2 at 1:1,000; Sigma), cardiac troponin T monoclonal antibody (MAb) (13-11 at 1:1,000; Invitrogen), and anti-ACE2 (AF933 at 1:50; R&D Systems). Rabbit anti-SARS-CoV nucleoprotein was used at a 1:5,000 dilution (generated by the Garcia-Sastre’s lab), and monoclonal anti-SARS-CoV nucleoprotein was used at a 1:1,000 dilution (MAb 1C7, a gift from Thomas Moran). All secondary antibodies were Alexa Fluor conjugated and purchased from Invitrogen. Cells were mounted with ProLong Gold with DAPI (4′,6-diamidino-2-phenylindole; Invitrogen) or with ProLong Gold (Invitrogen) and Hoechst stain (Sigma).

Cell diameters were measured using laser scanning microscopy (LSM) acquired images that were imported into ImageJ. We used ImageJ analysis by taking maximum-intensity projection of our images from the Zeiss LSM 880 and saving them as LSM files, to save the metadata for pixel-to-micrometer conversion. We had two individuals of the lab measure blind the cell diameter, as determined by troponin T staining, from the widest point for each cell. We had them count a minimum of 50 cells from 3 different CM lines per condition and averaged their measurements.

### RNA extraction, cDNA synthesis, and RT-qPCR.

Total RNAs from three human iPSC-derived CM cell lines were extracted with TRIzol (Invitrogen) using the chloroform-isopropranol extraction method according to the manufacturer’s instructions. RNA yield was determined using Qubit RNA BR assay kit (Life Technologies) and Qubit 3.0 fluorometer (Life Technologies). Total RNA (1 μg) was converted to cDNA using high-capacity cDNA reverse transcription kits (Applied Biosystems) and a Nexus GX2 master cycler. Quantitative PCR (qPCR) was performed using SYBR green (Quantabio) with custom primers (Fisher Scientific), and detection was achieved using the Applied Biosystems 7500 RT-PCR system. Levels of expression of target genes were normalized to the housekeeping gene coding for GADPH (glyceraldehyde-phosphate dehydrogenase). Fold expression was calculated using the mean for the threshold cycle number (*C_T_*) method from triplicate reactions per sample condition.

The primer sequences were as follows: for COVID-19, forward, 5′-GACCCCAAAATCAGCGAAAT-3′, and reverse, 5′-TCTGGTTACTGCCAGTTGAATCTG-3′; for GADPH, forward, 5′-GTGAAGGTCGGAGTCAACGG-3′, and reverse, 5′-TGACAAGCTTCCCGTTCTCA-3′; for cardiac troponin T, forward, 5′-TGATGACATCCACCGGAAGC-3′, and reverse, 5′-CTGCTTCTGGATGTAACCCCC-3′; for myosin light chain 4, forward, 5′-CCACGTCTCTCGGTTTCTTC-3′, and reverse, 5′-TAGGCTCAGGCTTCTTGGGA-3′; and for cardiac actin, forward, 5′-GTGCCAAGATGTGTGACGAC-3′, and reverse, 5′-TACCCACCATAACTCCCTGGT-3′.

### Troponin I ELISA.

Human troponin I ELISAs were performed using a commercially available kit (Thermo Fisher catalog no. EHTNNI3). We measured the amount of troponin released into the culture medium of the different CM lines tested. We treated the CM lines with SARS-CoV-2 in the presence or absence of IL-6 and/or IL-1β for 48 h. Typically 100 μL of cell culture medium was used for the assay. We followed the manufacturer’s protocols without any modification and quantified the amount of troponin based on the standard curve provided.

### Measurement of virus levels by TCID_50_ assay.

TCID_50_ assays were performed as described previously (54) In brief, Vero E6 cells were infected with serial 10-fold dilutions of the supernatants harvested from cells treated with IL-6 and/or IL-1β. On day 4 postinfection, cytopathic effect (CPE) was assessed by crystal violet staining, and TCID_50_ was calculated by the method of Reed and Muench (54).

### Reproducibility and statistics.

All experiments were carried out in at least 3 replicates, and the findings were reproduced in 3 to 4 separate experiments. All averages are from at least 3 separate experiments. Except for the CM cell beating experiments, we used three different cell lines in triplicate and imaged several different areas of each well for beating. This was done for two separate experiments for all 3 CM cell lines. Averages are from the two different experiments.

We used two-tailed analysis of variance (ANOVA) with Bonferroni corrections for multiple comparisons or *t* test analysis.

### Cardiac imaging.

Echocardiographic imaging was done as part of the clinical care of COVID-19 patients as determined by their physicians. The studies were performed using portable ultrasound machines: CX50 (Philips Medical Systems, Bothell, WA) and Vivid S70 (General Electric Medical Systems, Milwaukee, WI) (55, 56). Contrast-enhanced echocardiograms were performed using contrast-specific, low-mechanical-index settings after administration of ultrasonic enhancing agents: either Definity (perflutren lipid microsphere; Lantheus Medical Imaging) or Optison (perflutren protein type-A microspheres; GE Healthcare). Echocardiograms for COVID19-positive patients were performed in compliance with the following specific measures. Some studies were performed with transthoracic echocardiography (TTE) using the EPIQ echocardiography system (Philips North America, MA, USA). TTE studies were performed using a focused examination protocol according to American Society of Echocardiography recommendations for use of echocardiography in patients with confirmed COVID-19 infection.

### Data availability.

All data are available in the main text or in the supplemental material.
