# Supplementary material for: Functional Effects of Cardiomyocyte Injury in COVID-19
Source: J Virol. 2022 Jan 26;96(2):e01063-21. doi: 10.1128/JVI.01063-21 (PMC8791272; doi:10.1128/JVI.01063-21)
Supplement: Supplemental file 1 — Fig. S1 to S4; Tables S1 and S2; Legends to Movies S1 to S4 (available on author's website).. Download JVI.01063-21-s0001.pdf, PDF file, 1.8 MB [file jvi.01063-21-s0001.pdf]

Supplementary

Title: Functional effects of cardiomyocyte injury in COVID-19

Figure S1

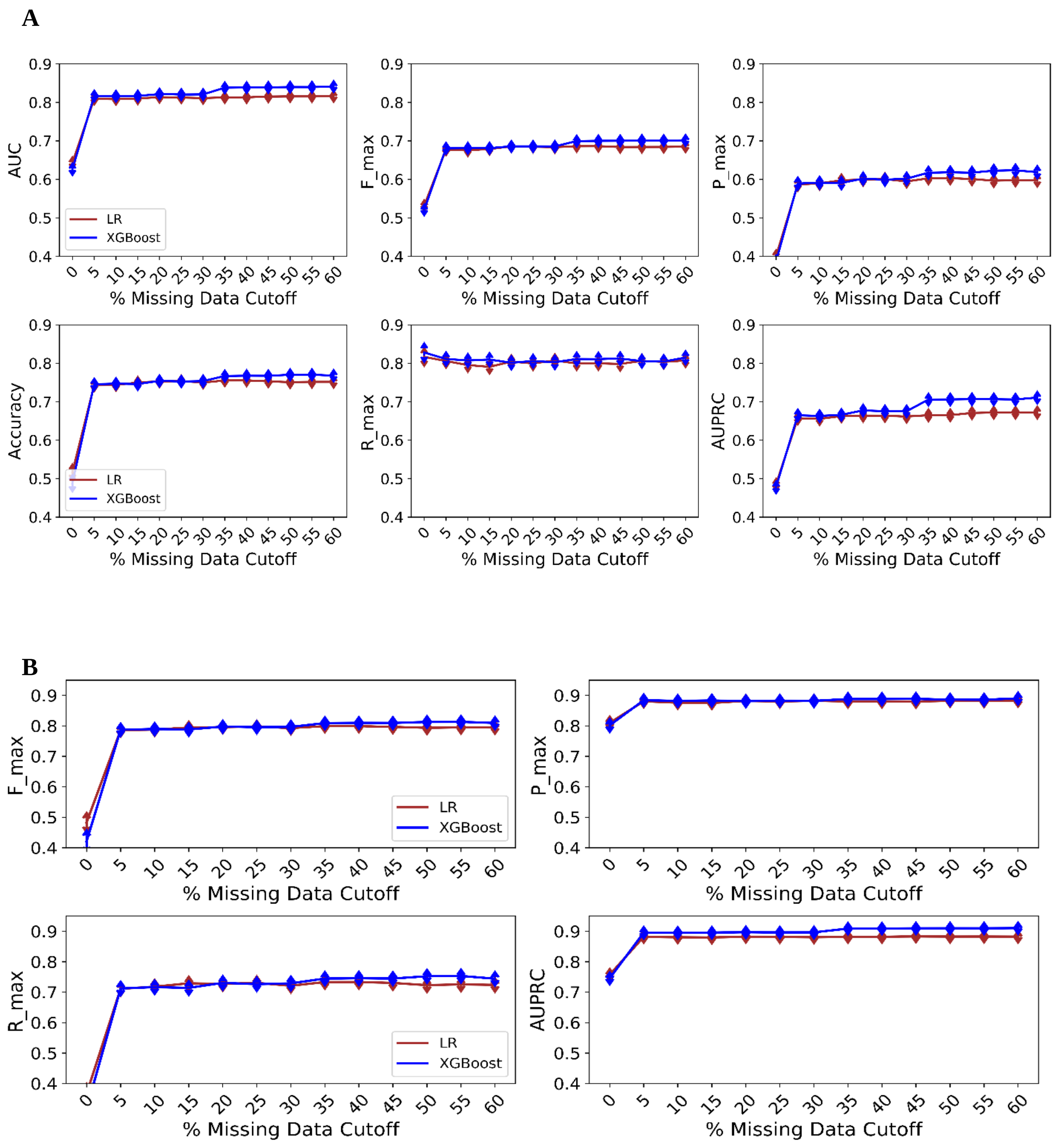

Figure S2

A

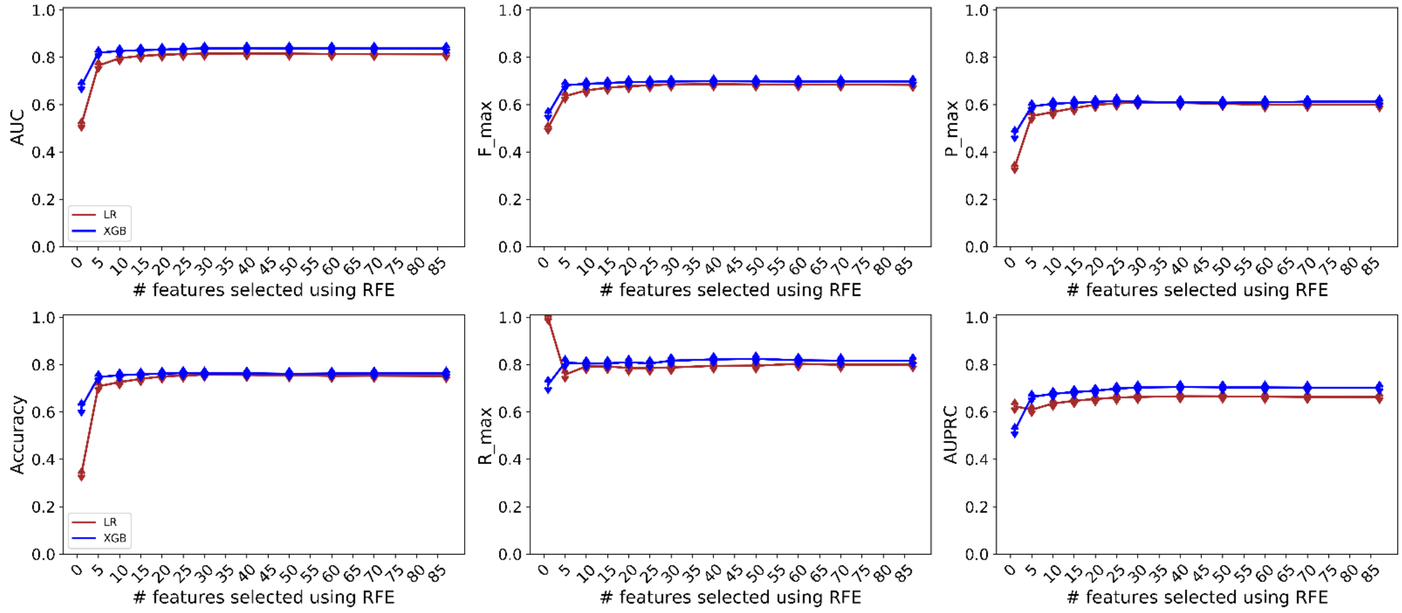

AUC
F_max
P_max
Accuracy
R_max
AUPRC
LR
XGB
features selected using RFE

B

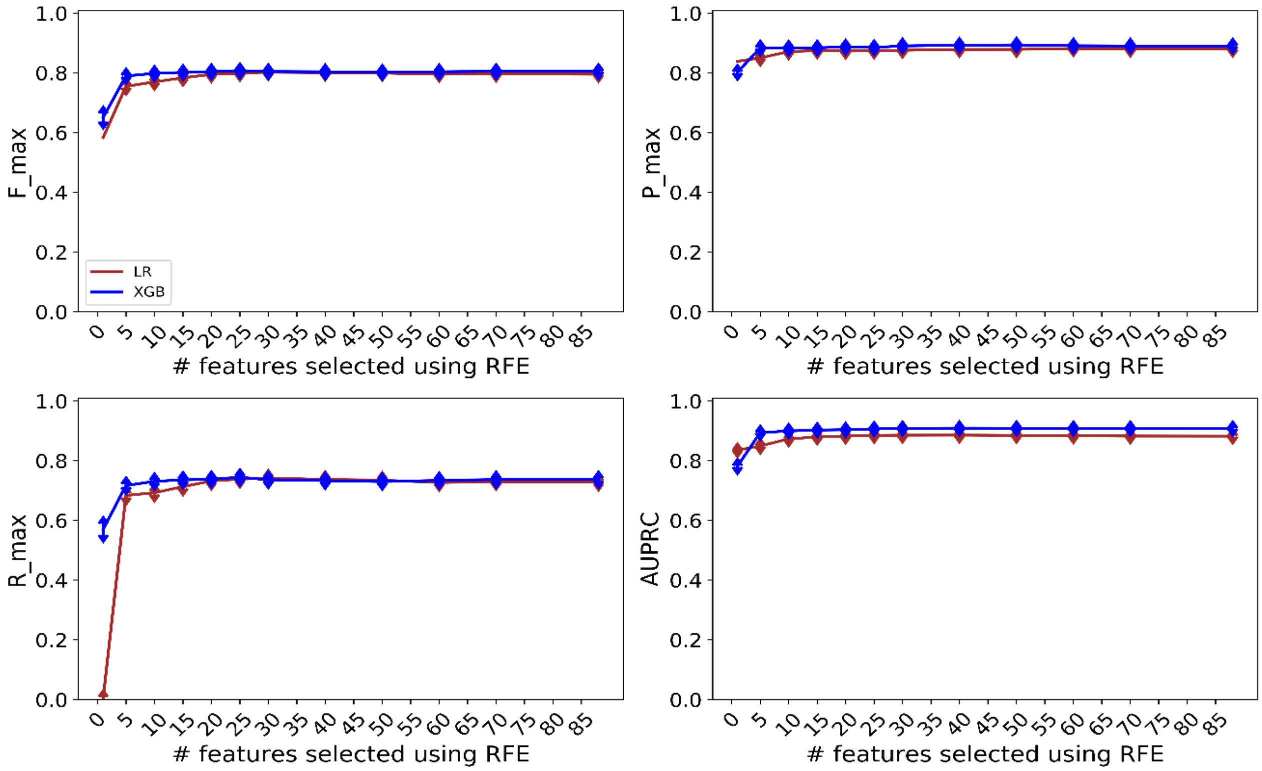

F_max
P_max
R_max
AUPRC
LR
XGB
features selected using RFE

Figure S3

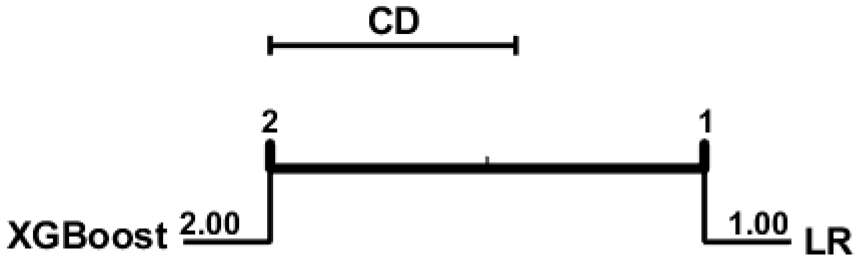

Supplementary Figure 4

A.

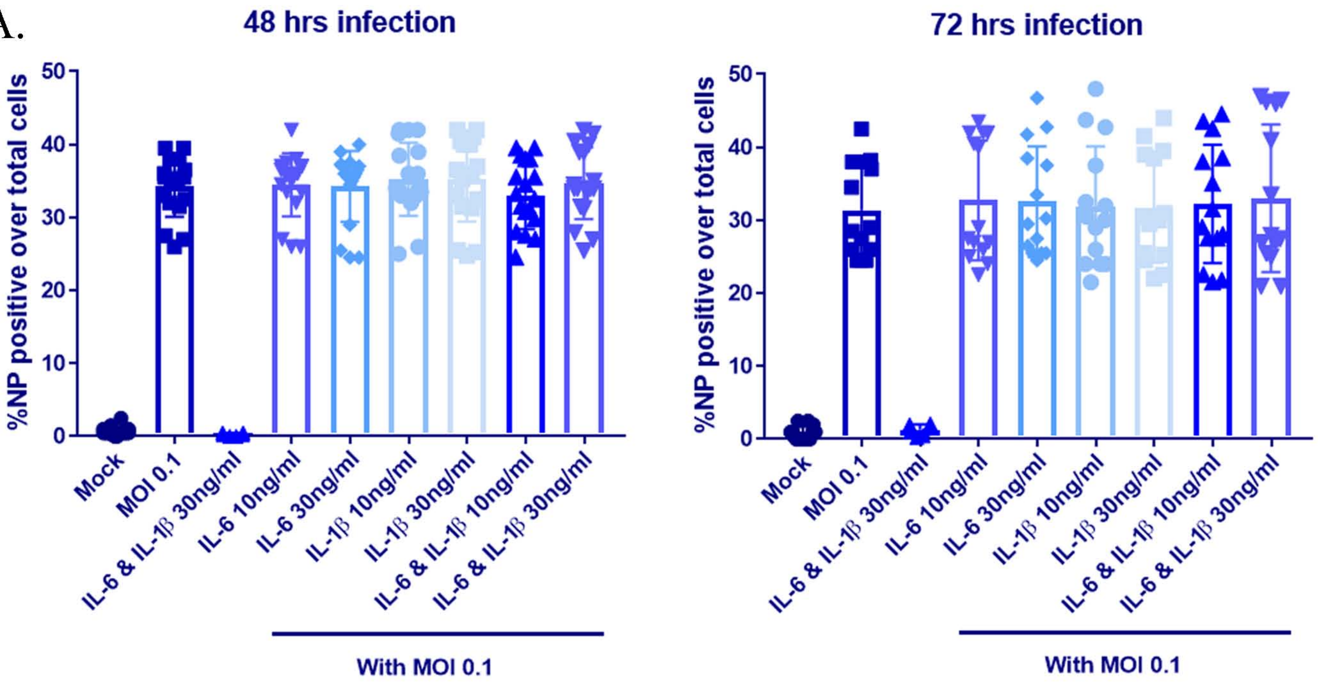

B.

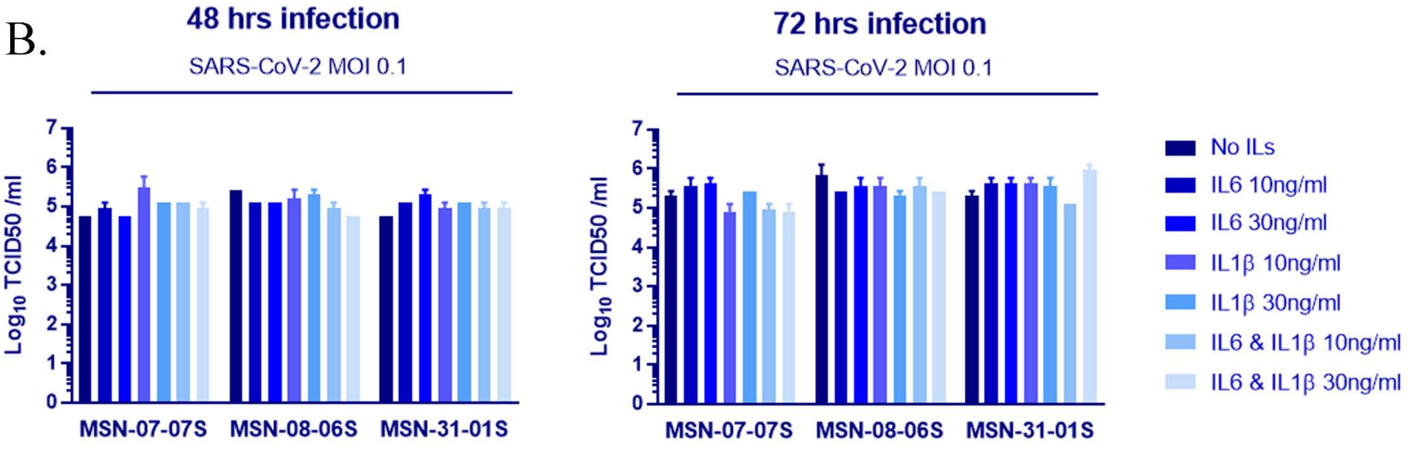

TABLE S1	Development Set (n = 3382) , 80 % of July 15th data			
**Variables (Continous Variables)**	**Total (n=3382)**	**Without Cardiac Disease (n = 2510)**	**With Cardiac Disease (n =872)**	**P-values***
AGE	68.39 (14.67)	66.69 (14.97)	73.27 (12.55)	3.04E-35
ALBUMIN	2.47 (0.71)	2.47 (0.72)	2.46 (0.67)	0.720983
ALT	148.88 (506.51)	155.88 (533.02)	128.9 (421.48)	0.134413
ANION_GAP	16.9 (5.56)	16.82 (5.66)	17.12 (5.29)	0.154926
AST	259.19 (1114.6)	271.19 (1192.09)	224.71 (853.44)	0.223857
BASOPHIL (%)	0.78 (0.88)	0.76 (0.86)	0.81 (0.93)	0.160497
BMI	28.48 (7.48)	28.45 (7.53)	28.54 (7.38)	0.761675
BRAIN NATRIURETIC PROTEIN	513.78 (1381.68)	308.59 (1012.71)	985.62 (1903.89)	8.21E-19
BUN	55.96 (44.61)	53.69 (44.76)	62.47 (43.54)	4.03E-07
C-REACTIVE PROTEIN	189.23 (121.25)	194.3 (122.47)	174.42 (116.42)	7.79E-05
CALCIUM	8.92 (0.9)	8.92 (0.9)	8.93 (0.88)	0.697778
CHLORIDE	109.97 (8.85)	110.07 (8.84)	109.67 (8.88)	0.252780
D-DIMER	5.47 (5.44)	5.63 (5.55)	5.0 (5.05)	0.005945
DIASTOLIC_BP	73.92 (15.17)	74.12 (14.81)	73.34 (16.15)	0.229716
DIASTOLIC_BP_MIN	48.36 (15.83)	49.13 (15.94)	46.16 (15.28)	2.03E-06
DIASTOL_BP_MAX	96.81 (17.48)	96.14 (17.3)	98.73 (17.85)	0.000283
EGFR	35.44 (28.9)	37.45 (30.48)	30.28 (23.63)	3.91E-11
EOSINOPHIL #	0.2 (0.39)	0.2 (0.42)	0.2 (0.31)	0.759916
EOSINOPHIL (%)	2.27 (3.35)	2.18 (3.3)	2.52 (3.46)	0.014025
FERRITIN	1139.88 (1865.7)	1169.3 (1913.5)	1053.59 (1716.18)	0.126327
GLUCOSE	274.63 (204.43)	272.5 (209.56)	280.74 (188.93)	0.281756
HCO3 VENOUS	26.53 (6.02)	26.29 (6.08)	27.19 (5.81)	0.000601
HEART_RATE	95.66 (20.67)	97.08 (20.09)	91.56 (21.77)	2.54E-10
HEART_RATE_MAX	120.21 (26.38)	119.84 (25.67)	121.3 (28.3)	0.189528
HEMOGLOBIN	10.56 (2.81)	10.71 (2.82)	10.14 (2.72)	1.11E-07
INR	1.57 (1.15)	1.49 (0.93)	1.8 (1.58)	1.30E-06
INTERLEUKIN-6	659.3 (7774.61)	771.59 (8948.25)	318.14 (839.28)	0.066442
LDH	751.96 (1181.87)	772.7 (1296.4)	689.58 (734.37)	0.037313
LYMPHOCYTE #	1.71 (2.37)	1.74 (2.55)	1.64 (1.78)	0.227937
LYMPHOCYTE (%)	21.06 (12.0)	21.14 (12.17)	20.83 (11.49)	0.495127
MCH	30.6 (2.87)	30.61 (2.83)	30.59 (2.97)	0.910013

MCHC	33.61 (1.47)	33.66 (1.49)	33.45 (1.41)	0.000131
MCV	93.22 (7.76)	93.03 (7.63)	93.76 (8.1)	0.018952
MEAN PLATELET VOLUME (MPV)	9.61 (1.68)	9.53 (1.69)	9.83 (1.64)	7.49E-06
MONOCYTE #	0.89 (0.65)	0.88 (0.68)	0.9 (0.53)	0.473918
MONOCYTE (%)	10.16 (5.27)	10.0 (5.16)	10.62 (5.53)	0.003678
NEUTROPHIL #	21.2 (94.88)	22.31 (109.32)	18.02 (22.19)	0.063645
NEUTROPHIL (%)	66.42 (15.79)	66.55 (15.81)	66.06 (15.73)	0.432086
O2 SATURATION VENOUS	79.36 (22.51)	79.4 (22.81)	79.26 (21.65)	0.882016
O2SAT_MIN	81.2 (20.03)	81.09 (20.3)	81.55 (19.23)	0.561668
O2_SAT	93.06 (7.6)	92.79 (7.82)	93.82 (6.86)	0.000385
PC02 VENOUS	49.31 (15.62)	49.0 (15.8)	50.17 (15.08)	0.078335
PH VENOUS	7.42 (0.08)	7.42 (0.08)	7.42 (0.08)	0.378739
PLATELET	180.5 (88.63)	183.73 (90.44)	171.23 (82.58)	0.000181
PO2 VENOUS	78.62 (55.71)	79.92 (56.74)	75.08 (52.69)	0.045458
POTASSIUM	5.31 (1.62)	5.3 (1.77)	5.36 (1.05)	0.238833
PROCALCITONIN	7.0 (36.47)	6.72 (35.22)	7.83 (40.02)	0.518561
PTT	53.95 (38.33)	53.19 (38.06)	56.09 (39.04)	0.098288
RBC COUNT	4.5 (0.77)	4.54 (0.75)	4.39 (0.83)	5.97E-06
RESPIRATORY_RATE	21.34 (5.89)	21.28 (5.77)	21.52 (6.22)	0.347297
RESP_RATE_MAX	33.91 (20.6)	33.64 (20.02)	34.7 (22.2)	0.227068
SERUM CREATININE	3.21 (3.86)	3.04 (3.61)	3.7 (4.48)	8.19E-05
SODIUM	142.84 (8.85)	142.8 (8.86)	142.95 (8.84)	0.681707
SYSTOLIC_BP	131.03 (25.6)	130.59 (24.99)	132.33 (27.27)	0.109561
SYSTOLIC_BP_MIN	89.03 (25.38)	89.76 (25.36)	86.92 (25.31)	0.005323
SYSTOL_BP_MAX	166.41 (26.8)	165.08 (26.97)	170.24 (25.94)	1.07E-06
TEMPERATURE	98.98 (1.97)	99.06 (2.11)	98.74 (1.48)	1.35E-06
TEMP_MAX	100.86 (2.08)	100.93 (2.17)	100.66 (1.8)	0.000517
TOTAL BILIRUBIN	1.25 (2.64)	1.26 (2.95)	1.22 (1.43)	0.653973
TOTAL_PROTEIN	7.2 (0.9)	7.23 (0.91)	7.11 (0.88)	0.001205
WBC	14.82 (10.73)	14.91 (10.85)	14.54 (10.37)	0.362529

Categorical Variables

**ACUTE KIDNEY INJURY, No (%)**				0.882585941

No	3128 (92.5)	2320 (92.4)	(808 (92.7)	
Yes	254 (7.5)	190 (7.6)	64 (7.3)	
**ACUTE MI, No (%)**				5.86E-09
No	3316 (98.0)	2482 (98.9)	(834 (95.6)	
Yes	66 (2.0)	28 (1.1)	38 (4.4)	
**ACUTE VENOUS THROMBOEMBOLISM, No (%)**				0.754975725
No	3354 (99.2)	2488 (99.1)	(866 (99.3)	
Yes	28 (0.8)	22 (0.9)	6 (0.7)	
**ALCOHOLIC NONALCOHOLIC LIVER DISEASE, No (%)**				0.336100166
No	3308 (97.8)	2451 (97.6)	(857 (98.3)	
Yes	74 (2.2)	59 (2.4)	15 (1.7)	
**ARDS, No (%)**				0.883856262
No	3358 (99.3)	2492 (99.3)	(866 (99.3)	
Yes	24 (0.7)	18 (0.7)	6 (0.7)	
**ASTHMA, No (%)**				7.20E-06
No	3225 (95.4)	2418 (96.3)	(807 (92.5)	
Yes	157 (4.6)	92 (3.7)	65 (7.5)	
**CANCER FLAG, No (%)**				0.001842591
No	3073 (90.9)	2304 (91.8)	(769 (88.2)	
Yes	309 (9.1)	206 (8.2)	103 (11.8)	
**CEREBRAL INFARCTION, No (%)**				0.395566942
No	3346 (98.9)	2486 (99.0)	(860 (98.6)	
Yes	36 (1.1)	24 (1.0)	12 (1.4)	
**CHRONIC KIDNEY DISEASE, No (%)**				3.25E-45
No	2898 (85.7)	2277 (90.7)	(621 (71.2)	
Yes	484 (14.3)	233 (9.3)	251 (28.8)	
**CHRONIC VIRAL HEPATITIS, No (%)**				0.933475607
No	3346 (98.9)	2483 (98.9)	(863 (99.0)	
Yes	36 (1.1)	27 (1.1)	9 (1.0)	
**COPD, No (%)**				3.53E-22
No	3205 (94.8)	2434 (97.0)	(771 (88.4)	
Yes	177 (5.2)	76 (3.0)	101 (11.6)	
**CROHNS DISEASE, No (%)**				0.792032466

No	3375 (99.8)	2505 (99.8)	(870 (99.8)	
Yes	7 (0.2)	5 (0.2)	2 (0.2)	
**DIABETES, No (%)**				9.52E-43
No	2484 (73.4)	1998 (79.6)	(486 (55.7)	
Yes	898 (26.6)	512 (20.4)	386 (44.3)	
**ENCOUNTER TYPE, No (%)**				0.888291625
Inpatient	3147 (93.1)	2337 (93.1)	810 (92.9)	
Other	235 (6.9)	173 (6.9)	62 (7.1)	
**HIV FLAG, No (%)**				0.940816165
No	3317 (98.1)	2461 (98.0)	856 (98.2)	
Yes	65 (1.9)	49 (2.0)	16 (1.8)	
**HYPERTENSION, No (%)**				7.58E-93
No	1991 (58.9)	1734 (69.1)	257 (29.5)	
Yes	1391 (41.1)	776 (30.9)	615 (70.5)	
**ICU, No (%)**				0.323233683
No	2429 (71.8)	1813 (72.2)	616 (70.6)	
Yes	707 (20.9)	514 (20.5)	193 (22.1)	
**INPATIENT NON ICU, No (%)**				0.003910682
No	573 (16.9)	453 (18.0)	120 (13.8)	
Yes	2563 (75.8)	1874 (74.7)	689 (79.0)	
**INTRACEREBRAL HEMORRHAGE, No (%)**				0.543395076
No	3378 (99.9)	2506 (99.8)	872 (100.0)	
Yes	4 (0.1)	4 (0.2)	0 (0.0)	
**OBESITY, No (%)**				1.45E-12
No	3100 (91.7)	2351 (93.7)	749 (85.9)	
Yes	282 (8.3)	159 (6.3)	123 (14.1)	
**OBSTRUCTIVE SLEEP APNEA, No (%)**				3.78E-06
No	3302 (97.6)	2469 (98.4)	833 (95.5)	
Yes	80 (2.4)	41 (1.6)	39 (4.5)	
**RACE ETHNICITY COMBINED, No (%)**				5.13E-05
ASIAN	157 (4.6)	118 (4.7)	39 (4.5)	
BLACK OR AFRICAN-AMERICAN	918 (27.1)	694 (27.6)	224 (25.7)	
HISPANIC	923 (27.3)	686 (27.3)	237 (27.2)	

WHITE	798 (23.6)	538 (21.4)	260 (29.8)	
OTHER	457 (13.5)	367 (14.6)	90 (10.3)	
NATIVE HAWAIIAN OR PACIFIC ISLANDER	1 (0.0)	1 (0.0)	0 (0.0)	
AMERICAN INDIAN OR ALASKA NATIVE	1 (0.0)	1 (0.0)	0 (0.0)	
**SEX, No (%)**				0.77155788
MALE	1993 (58.9)	1475 (58.8)	518 (59.4)	
FEMALE	1389 (41.1)	1035 (41.2)	354 (40.6)	
**SMOKING , No (%)**				9.65E-13
NEVER	1701 (50.3)	1266 (50.4)	435 (49.9)	
Current	153 (4.5)	117 (4.7)	36 (4.1)	
Past	807 (23.9)	483 (19.2)	324 (37.2)	
PASSIVE	2 (0.1)	1 (0.0)	1 (0.1)	
**ULCERATIVE COLITIS, No (%)**				0.925026688
No	3369 (99.6)	2501 (99.6)	868 (99.5)	
Yes	13 (0.4)	9 (0.4)	4 (0.5)	

*P-value from student t-test for continuous variables and chisquare test for categorical variables

TABLE S1	Test Set (n = 846), 20% of 15th July data			
**Variables (Continous Variables)**	**Total (n=846)**	**Without Cardiac Disease (n = 653)**	**With Cardiac Disease (n = 193)**	**P-values***
AGE	67.47 (15.71)	65.88 (16.38)	72.85 (11.76)	1.44E-10
ALBUMIN	2.44 (0.69)	2.45 (0.69)	2.42 (0.69)	0.616719
ALT	128.37 (517.97)	119.3 (540.1)	158.17 (437.36)	0.310716
ANION_GAP	16.79 (5.1)	16.52 (5.09)	17.7 (5.04)	0.004923
AST	224.13 (1291.02)	218.91 (1427.38)	241.51 (661.54)	0.763038
BASOPHIL (%)	0.87 (2.63)	0.88 (2.95)	0.81 (0.93)	0.565992
BMI	28.83 (7.46)	29.32 (7.67)	27.4 (6.63)	0.001363
BRAIN NATRIURETIC PROTEIN	513.83 (1279.54)	294.63 (764.67)	1120.09 (2016.66)	1.29E-06
BUN	55.54 (44.87)	53.05 (45.01)	63.96 (43.43)	0.002619
C-REACTIVE PROTEIN	195.09 (127.66)	204.24 (130.53)	163.3 (111.79)	9.26E-05
CALCIUM	8.96 (0.82)	8.95 (0.83)	9.0 (0.79)	0.424453
CHLORIDE	110.11 (8.65)	110.08 (8.58)	110.19 (8.87)	0.876153
D-DIMER	5.59 (5.45)	5.93 (5.62)	4.35 (4.57)	0.000498
DIASTOLIC_BP	73.54 (15.2)	74.06 (14.49)	71.77 (17.35)	0.108707
DIASTOLIC_BP_MIN	48.33 (15.59)	49.14 (15.85)	45.6 (14.35)	0.004165
DIASTOL_BP_MAX	96.98 (17.73)	96.87 (17.51)	97.37 (18.51)	0.743188
EGFR	35.41 (28.73)	37.87 (29.86)	27.78 (23.41)	5.99E-06
EOSINOPHIL #	0.22 (0.49)	0.23 (0.53)	0.22 (0.3)	0.798222
EOSINOPHIL (%)	2.56 (3.53)	2.47 (3.45)	2.87 (3.81)	0.191102
FERRITIN	1236.76 (2344.07)	1039.09 (1330.44)	1915.9 (4216.83)	0.009662
GLUCOSE	274.94 (195.55)	271.99 (197.48)	284.9 (189.05)	0.411366
HCO3 VENOUS	26.38 (5.33)	26.35 (5.19)	26.47 (5.8)	0.811889
HEART_RATE	97.18 (20.33)	98.92 (20.08)	91.31 (20.15)	1.01E-05
HEART_RATE_MAX	120.38 (24.43)	120.44 (23.97)	120.19 (25.98)	0.906720
HEMOGLOBIN	10.54 (2.78)	10.67 (2.8)	10.1 (2.71)	0.010942
INR	1.51 (0.96)	1.44 (0.92)	1.74 (1.04)	0.001209
INTERLEUKIN-6	373.43 (994.97)	385.3 (1073.65)	330.66 (637.93)	0.530598
LDH	716.0 (927.29)	708.12 (960.19)	743.53 (804.15)	0.647665
LYMPHOCYTE #	1.7 (1.35)	1.7 (1.36)	1.67 (1.34)	0.808482
LYMPHOCYTE (%)	21.65 (12.33)	21.71 (12.25)	21.43 (12.65)	0.787784
MCH	30.36 (2.79)	30.31 (2.75)	30.5 (2.92)	0.430252

MCHC	33.62 (1.4)	33.7 (1.37)	33.34 (1.44)	0.002191
MCV	92.36 (7.5)	91.96 (7.34)	93.71 (7.87)	0.006400
MEAN PLATELET VOLUME (MPV)	9.58 (1.6)	9.5 (1.61)	9.84 (1.52)	0.008070
MONOCYTE #	0.9 (0.55)	0.91 (0.54)	0.9 (0.57)	0.902433
MONOCYTE (%)	10.15 (4.75)	10.08 (4.73)	10.4 (4.84)	0.420691
NEUTROPHIL #	18.81 (22.93)	19.11 (23.17)	17.8 (22.13)	0.478494
NEUTROPHIL (%)	65.89 (15.73)	65.89 (16.1)	65.87 (14.41)	0.986668
O2 SATURATION VENOUS	79.93 (22.03)	79.72 (21.94)	80.68 (22.38)	0.642645
O2SAT_MIN	82.18 (18.58)	82.48 (18.27)	81.2 (19.6)	0.431577
O2_SAT	93.37 (7.18)	92.91 (7.65)	94.92 (4.99)	3.42E-05
PC02 VENOUS	48.71 (14.47)	48.34 (14.34)	49.98 (14.85)	0.221491
PH VENOUS	7.42 (0.07)	7.43 (0.07)	7.42 (0.07)	0.165882
PLATELET	181.56 (85.45)	186.18 (85.81)	166.03 (82.55)	0.003407
PO2 VENOUS	78.07 (55.87)	75.94 (54.1)	85.32 (61.15)	0.096001
POTASSIUM	5.28 (1.06)	5.24 (1.04)	5.43 (1.11)	0.036127
PROCALCITONIN	9.54 (43.63)	10.24 (46.62)	7.11 (31.06)	0.331820
PTT	53.48 (36.92)	51.81 (35.49)	58.96 (40.93)	0.058061
RBC COUNT	4.57 (0.78)	4.62 (0.74)	4.39 (0.86)	0.000913
RESPIRATORY_RATE	21.61 (5.83)	21.8 (5.98)	20.98 (5.25)	0.075160
RESP_RATE_MAX	33.53 (19.68)	33.58 (18.94)	33.38 (22.06)	0.911150
SERUM CREATININE	3.2 (3.6)	2.94 (3.41)	4.07 (4.07)	0.000527
SODIUM	142.73 (8.23)	142.68 (8.28)	142.9 (8.07)	0.754595
SYSTOLIC_BP	130.83 (26.08)	130.65 (25.09)	131.48 (29.28)	0.729811
SYSTOLIC_BP_MIN	89.25 (24.49)	90.7 (24.88)	84.36 (22.53)	0.001073
SYSTOL_BP_MAX	165.42 (26.68)	165.22 (26.46)	166.11 (27.44)	0.693991
TEMPERATURE	99.04 (2.78)	99.2 (1.76)	98.47 (4.8)	0.044077
TEMP_MAX	100.93 (2.09)	101.03 (2.19)	100.61 (1.72)	0.006975
TOTAL BILIRUBIN	1.17 (1.59)	1.15 (1.73)	1.22 (1.02)	0.486184
TOTAL_PROTEIN	7.26 (0.92)	7.24 (0.87)	7.3 (1.09)	0.538424
WBC	14.66 (9.44)	14.8 (9.68)	14.2 (8.59)	0.407910

Categorical Variables

**ACUTE KIDNEY INJURY, No (%)**				0.62476289

No	789 (93.3)	611 (93.6)	178 (92.2)	
Yes	57 (6.7)	42 (6.4)	15 (7.8)	
**ACUTE MI, No (%)**				0.13858252
No	832 (98.3)	645 (98.8)	187 (96.9)	
Yes	14 (1.7)	8 (1.2)	6 (3.1)	
**ACUTE VENOUS THROMBOEMBOLISM, No (%)**				0.32113486
No	839 (99.2)	646 (98.9)	193 (100.0)	
Yes	7 (0.8)	7 (1.1)	0 (0.0)	
**ALCOHOLIC NONALCOHOLIC LIVER DISEASE, No (%)**				0.11837316
No	812 (96.0)	631 (96.6)	181 (93.8)	
Yes	34 (4.0)	22 (3.4)	12 (6.2)	
**ARDS, No (%)**				0.65864418
No	837 (98.9)	645 (98.8)	192 (99.5)	
Yes	9 (1.1)	8 (1.2)	1 (0.5)	
**ASTHMA, No (%)**				0.00750029
No	798 (94.3)	624 (95.6)	174 (90.2)	
Yes	48 (5.7)	29 (4.4)	19 (9.8)	
**CANCER FLAG, No (%)**				0.00771183
No	774 (91.5)	607 (93.0)	167 (86.5)	
Yes	72 (8.5)	46 (7.0)	26 (13.5)	
**CEREBRAL INFARCTION, No (%)**				0.65864418
No	837 (98.9)	645 (98.8)	192 (99.5)	
Yes	9 (1.1)	8 (1.2)	1 (0.5)	
**CHRONIC KIDNEY DISEASE, No (%)**				5.04E-20
No	706 (83.5)	587 (89.9)	119 (61.7)	
Yes	140 (16.5)	66 (10.1)	74 (38.3)	
**CHRONIC VIRAL HEPATITIS, No (%)**				0.7212213
No	837 (98.9)	647 (99.1)	190 (98.4)	
Yes	9 (1.1)	6 (0.9)	3 (1.6)	
**COPD, No (%)**				0.00193523
No	817 (96.6)	638 (97.7)	179 (92.7)	
Yes	29 (3.4)	15 (2.3)	14 (7.3)	
**CROHNS DISEASE, No (%)**				0.07826238

No	844 (99.8)	653 (100.0)	191 (99.0)	
Yes	2 (0.2)	0 (0.0)	2 (1.0)	
**DIABETES, No (%)**				3.20E-12
No	626 (74.0)	521 (79.8)	105 (54.4)	
Yes	220 (26.0)	132 (20.2)	[88 (45.6)	
**ENCOUNTER TYPE, No (%)**				0.87109763
Inpatient	789 (93.3)	610 (93.4)	179 (92.7)	
Other	57 (6.7)	43 (6.6)	14 (7.3)	
**HIV FLAG, No (%)**				0.65864418
No	837 (98.9)	645 (98.8)	192 (99.5)	
Yes	9 (1.1)	8 (1.2)	1 (0.5)	
**HYPERTENSION, No (%)**				1.34E-20
No	505 (59.7)	446 (68.3)	59 (30.6)	
Yes	341 (40.3)	207 (31.7)	134 (69.4)	
**ICU, No (%)**				0.74146061
No	598 (70.7)	460 (70.4)	138 (71.5)	
Yes	190 (22.5)	149 (22.8)	41 (21.2)	
**INPATIENT NON ICU, No (%)**				0.35425853
No	144 (17.0)	116 (17.8)	28 (14.5)	
Yes	644 (76.1)	493 (75.5)	151 (78.2)	
**INTRACEREBRAL HEMORRHAGE, No (%)**				0.94118182
No	844 (99.8)	652 (99.8)	192 (99.5)	
Yes	2 (0.2)	1 (0.2)	1 (0.5)	
**OBESITY, No (%)**				0.68862702
No	772 (91.3)	594 (91.0)	178 (92.2)	
Yes	74 (8.7)	59 (9.0)	15 (7.8)	
**OBSTRUCTIVE SLEEP APNEA, No (%)**				0.51927591
No	827 (97.8)	640 (98.0)	187 (96.9)	
Yes	19 (2.2)	13 (2.0)	6 (3.1)	
**RACE ETHNICITY COMBINED, No (%)**				3.70E-05
ASIAN	47 (5.6)	34 (5.2)	13 (6.7)	
BLACK OR AFRICAN-AMERICAN	221 (26.1)	178 (27.3)	43 (22.3)	
HISPANIC	236 (27.9)	196 (30.0)	40 (20.7)	

WHITE	169 (20.0)	108 (16.5)	61 (31.6)	
OTHER	139 (16.4)	114 (17.5)	25 (13.0)	
NATIVE HAWAIIAN OR PACIFIC ISLANDER	0 (0.0)	0 (0.0)	0 (0.0)	
AMERICAN INDIAN OR ALASKA NATIVE	0 (0.0)	0 (0.0)	0 (0.0)	
**SEX, No (%)**				0.75413949
MALE	497 (58.7)	386 (59.1)	111 (57.5)	
FEMALE	349 (41.3)	267 (40.9)	82 (42.5)	
**SMOKING , No (%)**				0.00115863
NEVER	439 (51.9)	345 (52.8)	94, 48.7	
Current	37 (4.4)	26 (4.0)	11, 5.7	
Past	187 (22.1)	121 (18.5)	66, 34.2	
PASSIVE	0 (0.0)	0 (0.0)	0 (0.0)	
**ULCERATIVE COLITIS, No (%)**				
No	846 (100.0)	653 (100.0)	193 (100.0)	1
Yes	0 (0.0)	0 (0.0)	0 (0.0)	

*P-value from student t-test for continuous variables and chisquare test for categorical variables

**TABLE S2- Variables (Continous Variables)**	**Development Set (n = 3382)**	**Test Set (n = 846)**
AGE	0	0
ALBUMIN	86	27
ALT	92	27
ANION_GAP	8	6
AST	137	37
BASOPHIL (%)	35	22
BMI	520	136
BRAIN NATRIURETIC PROTEIN	980	251
BUN	7	5
C-REACTIVE PROTEIN	468	116
CALCIUM	5	5
CHLORIDE	4	3
D-DIMER	661	162
DIASTOLIC_BP	230	53
DIASTOLIC_BP_MIN	142	30
DIASTOL_BP_MAX	142	30
EGFR	565	137
EOSINOPHIL #	79	22
EOSINOPHIL (%)	35	15
FERRITIN	499	123
GLUCOSE	4	5
HCO3 VENOUS	772	173
HEART_RATE	209	46
HEART_RATE_MAX	143	30
HEMOGLOBIN	7	4
INR	832	206
INTERLEUKIN-6	1589	404
LDH	617	163
LYMPHOCYTE #	79	22
LYMPHOCYTE (%)	29	13
MCH	7	4
MCHC	7	4
MCV	7	4
MEAN PLATELET VOLUME (MPV)	21	9
MONOCYTE #	79	22
MONOCYTE (%)	29	13
NEUTROPHIL #	11	9
NEUTROPHIL (%)	29	13
O2 SATURATION VENOUS	794	189
O2SAT_MIN	211	46
O2_SAT	211	46
PC02 VENOUS	671	158
PH VENOUS	671	158
PLATELET	11	4

PO2 VENOUS	852	204
POTASSIUM	17	5
PROCALCITONIN	642	160
PTT	866	219
RBC COUNT	7	4
RESPIRATORY_RATE	220	50
RESP_RATE_MAX	146	33
SERUM CREATININE	7	5
SODIUM	427	95
SYSTOLIC_BP	230	53
SYSTOLIC_BP_MIN	142	30
SYSTOL_BP_MAX	142	30
TEMPERATURE	209	46
TEMP_MAX	209	46
TOTAL BILIRUBIN	86	29
TOTAL_PROTEIN	104	32
WBC	8	4

Categorical Variables

ACUTE KIDNEY INJURY	0	0
ACUTE MI	0	0
ACUTE VENOUS THROMBOEMBOLISM	0	0
ALCOHOLIC NONALCOHOLIC LIVER DISEASE	0	0
ARDS	0	0
ASTHMA	0	0
CANCER FLAG	0	0
CEREBRAL INFARCTION	0	0
CHRONIC KIDNEY DISEASE	0	0
CHRONIC VIRAL HEPATITIS	0	0
COPD	0	0
CROHNS DISEASE	0	0
DIABETES	0	0
ENCOUNTER TYPE	0	0
HIV FLAG	0	0
HYPERTENSION	0	0
ICU	246	58
INPATIENT NON ICU	246	58
INTRACEREBRAL HEMORRHAGE	0	0
OBESITY	0	0
OBSTRUCTIVE SLEEP APNEA	0	0
RACE ETHNICITY COMBINED	127	34
SEX	0	0
SMOKING	719	183
ULCERATIVE COLITIS	0	0

Supplementary Figure S1. Data driven assessment to determine optimal manner dealing with missing data during the model development process. To evaluate the optimal manner in which to deal with features with missing data, different cutoffs were taken in 5% increments (0% to 60%) of missing data and using the training set, the mean-mode imputation method was evaluated (mean for continuous features, mode for categorical features). Two different classifiers (LR, XGBoost) were then trained at each of the percent missing data cutoffs on the training dataset and validated on the 20% validation dataset with subsequent evaluation by six different metrics (AUC, Accuracy, F_max, R_max, P_max, AUPRC) in order to assess model performance at each cutoff. Each model was run 100 times for each missing data cutoff and the average of the performance was plotted for each metric for the **A.)** Troponin level more than 0.09 and **B.)** Troponin level less than equal to 0.09 (AUC and Accuracy are not presented as these are class independent metrics). Data points show the average AUC score for each candidate algorithm and missing value level, with error bars shown by whiskers. AUC – area under the receiver operating characteristic curve; AUPRC – area under the precision recall curve; LR – logistic regression; P_max – precision maximum; R_max – recall maximum; F_max – f1-score which is harmonic mean of precision and recall ;XGBoost – extreme gradient boosting.

Supplementary Figure S2. Results of recursive feature elimination during the model development phase using preoperative only dataset. Recursive feature elimination is a feature selection method that fits a model and removes the weakest feature (or features) until the pre-specified number of features is reached. Various incremental percentages (0% to 100%) of the overall number of features (100% features = 86 features) were assessed. Two different classifiers (LR, XGBoost) were then trained at each of the percent missing data cutoffs on the training dataset 75% and validated on the 25% validation dataset with subsequent evaluation by six different metrics (AUC, Accuracy, Fmax, Rmax, Pmax, AUPRC) in order to assess model performance at each cutoff. Each model was run 100 times for each missing data cutoff and the average of the performance was plotted for each metric for the **A.)** Troponin level more than 0.09 and **B.)** Troponin level less than equal to 0.09 (AUC and Accuracy are not presented as these are class independent metrics). Data points show the average AUC score for each candidate algorithm and

missing value level, with error bars shown by whiskers. AUC – area under the receiver operating characteristic curve; AUPRC – area under the precision recall curve; LR – logistic regression P_max – precision maximum; R_max – recall maximum; F_max – f1-score which is harmonic mean of precision and recall; XGBoost – extreme gradient boosting.

Supplementary Figure S3. The statistical comparison of two classification algorithms performances in the form of Critical Difference (CD) plots: Classification algorithms, represented by (vertical + horizontal) lines, are displayed from left to right in terms of the average rank obtained by their resultant models in each of the 100 simulations on validation data set. The classifiers producing statistically significantly different performance because both classifiers are not connected by horizontal lines. These results show that the XGBoost is the best performer overall. The CD plots were drawn using open-source Matlab code.

Supplementary Figure S4. ILs do not increase the infectivity of SARS-CoV-2 as determined by NP-positive staining and shedding of virus into culture media.

(A) We counted the numbers of cells stained by N protein antibody as a measure of infectivity, using either IL-6 and/or IL-1β at 10 or 30ng/ml. Each point is representative from one well of a 96 well plate with 10,000 CM cells. The bar graph is the average of four different CM lines measuring viral NP protein positive cells over total number of cells. Counting was done in an automated fashion using the InCell Analyzer. No statistical difference was determined between infection alone or in any combination of ILs tested.

(B) ILs do not increase the percentage of CM cells infected by SARS-CoV-2. To confirm that CM were being productively infected and shedding SARS-CoV-2 into the culture media, we collected supernatants from 3 different CM lines 48 and 72hrs post-infection, with or without ILs and performed a TCID50 plaque assay. We observed that CM lines are infected and the level of infection, as assessed by virus release in to culture medium did not increase with the addition of IL-6 and IL-1β, there was no statistical difference detected for any condition when comparing to No ILs.

Table S1. Descriptive characteristics of the overall development (80%) dataset, and 20% test dataset. Characteristic table for development set and test set are presented. Both data sets further divided patients with and without cardiac disease. For continuous variables shown with mean and standard deviation in parentheses. Counts and percentages (in parentheses) are presented for categorical variables.

Table S2: Missing value number of development set and test set.

Data acquired from MSDW and many features have missing values which are listed in this table. It represents the number of missing values in development set and test set that are imputed by missing value imputation techniques in the model.

List of Supplementary Movies

https://iyengarlab.org/ - All movies are provided on our lab webpage.

Movie S1 – Live cell beating of hIPSC cardiomyocytes at 48hrs post infection with SARS-CoV-2. The movie has been sped up 6X and displays MSN08-06S CM line beating in the BSL3 in the following order: CM line MSN08-06S (8-48h): MOCK, Infection only at MOI 0.1, Interleukins (ILs) only (IL-6 and IL-1β each at 30ng/ml) and ILs with Infection.

All patients are confirmed to be COVID-19 positive and no history of cardiac disease. Movie 2 has normal LVEF, while 3 and 4 have newly reduced LVEF<51.

Movie S2 - Normal demonstrates an apical 4-chamber view (after administration of an ultrasonic enhancing agent) from a transthoracic echocardiogram. The findings are consistent with preserved left ventricular ejection fraction and no regional wall motion abnormalities. The end-diastolic and end-systolic volumes are 104ml and 35ml, respectively with a left ventricular ejection fraction of 66%.

Movie S3 – Regional Wall Motion Abnormality (RWMA) demonstrates an apical 3-chamber view (after administration of an ultrasonic enhancing agent) from a transthoracic

echocardiogram. The findings are consistent with basal and mid infero-lateral wall hypokinesis despite preserved left ventricular ejection fraction. The end-diastolic and end-systolic volumes are 102ml and 50ml, respectively with a left ventricular ejection fraction of 51%

Movie S4 – Diffuse demonstrates an apical 4-chamber view (after administration of an ultrasonic enhancing agent) from a transthoracic echocardiogram. The findings are consistent with diffuse left ventricular wall hypokinesis and mildly decreased left ventricular ejection fraction. The end-diastolic and end-systolic volumes are 160ml and 90ml, respectively with a left ventricular ejection fraction of 44%.
